# FDG-PET-based neural correlates of Addenbrooke’s cognitive examination III scores in Alzheimer’s disease and frontotemporal degeneration

**DOI:** 10.3389/fpsyg.2023.1273608

**Published:** 2023-11-16

**Authors:** María Nieves Cabrera-Martín, Pedro Nespral, Maria Valles-Salgado, Pablo Bascuñana, Cristina Delgado-Alonso, Alfonso Delgado-Álvarez, Lucía Fernández-Romero, Juan Ignacio López-Carbonero, María Díez-Cirarda, María José Gil-Moreno, Jorge Matías-Guiu, Jordi A. Matias-Guiu

**Affiliations:** ^1^Department of Neurology, San Carlos Institute for Health Research (IdISSC), Universidad Complutense, Madrid, Spain; ^2^Department of Nuclear Medicine, San Carlos Institute for Health Research (IdISSC), Universidad Complutense, Madrid, Spain

**Keywords:** Alzheimer’s disease, frontotemporal dementia, neuropsychological assessment, Addenbrooke’s cognitive examination, positron emission tomography

## Abstract

**Introduction:**

The Addenbrooke’s Cognitive Examination III (ACE-III) is a brief test useful for neuropsychological assessment. Several studies have validated the test for the diagnosis of Alzheimer’s disease (AD) and frontotemporal dementia (FTD). In this study, we aimed to examine the metabolic correlates associated with the performance of ACE-III in AD and behavioral variant FTD.

**Methods:**

We enrolled 300 participants in a cross-sectional study, including 180 patients with AD, 60 with behavioral FTD (bvFTD), and 60 controls. An ^18^F-Fluorodeoxyglucose positron emission tomography study was performed in all cases. Correlation between the ACE-III and its domains (attention, memory, fluency, language, and visuospatial) with the brain metabolism was estimated.

**Results:**

The ACE-III showed distinct neural correlates in bvFTD and AD, effectively capturing the most relevant regions involved in these disorders. Neural correlates differed for each domain, especially in the case of bvFTD. Lower ACE-III scores were associated with more advanced stages in both disorders. The ACE-III exhibited high discrimination between bvFTD vs. HC, and between AD vs. HC. Additionally, it was sensitive to detect hypometabolism in brain regions associated with bvFTD and AD.

**Conclusion:**

Our study contributes to the knowledge of the brain regions associated with ACE-III, thereby facilitating its interpretation, and highlighting its suitability for screening and monitoring. This study provides further validation of ACE-III in the context of AD and FTD.

## Introduction

The Addenbrooke’s Cognitive Examination III (ACE-III) is a screening cognitive test developed for diagnosing cognitive disorders, particularly frontotemporal dementia (FTD) and Alzheimer’s disease (AD) (Hsieh et al., 2013). Initially, it was validated as a screening tool for cognitive impairment in FTD and AD, but subsequently, it has shown high utility for the diagnosis of amnestic mild cognitive impairment, mild dementia of several types, early-onset dementia and dementia in the elderly (Jubb and Evans, 2015; Matías-Guiu et al., 2015, 2017; Elamin et al., 2016). Furthermore, it has been validated in several languages and settings (Charerboon et al., 2016; Wang et al., 2017). ACE-III belongs to a new generation of brief cognitive tests designed to enhance the diagnostic capabilities of the Mini-Mental State Examination (Matias-Guiu et al., 2017). Importantly, ACE-III plays a dual function in cognitive examination: firstly, to screen for cognitive disorders; and, secondly, to obtain a cognitive profile (Matías-Guiu et al., 2016). The assessment covers five cognitive domains, and the strong correlations observed between these domains and standardized neuropsychological tests suggest that ACE-III could offer valuable information for differentiating between neurodegenerative diseases (Matías-Guiu et al., 2017; So et al., 2018; Zarrella et al., 2023).

The similarities and differences in the cognitive profile between bvFTD and AD is a matter of debate. While bvFTD is characterized by executive function impairment and relatively preserved episodic memory and visuospatial function (Rascovsky et al., 2011), these distinctions are not absolute, as some patients with bvFTD may exhibit cognitive profiles similar to those seen in AD. It’s worth noting that executive function is also affected in AD, and memory can be impaired in bvFTD. However, cognitive tests remain crucial for the diagnosis of these disorders across various levels of the healthcare system.

One of the main challenges in neuropsychological assessment is the interpretation of the findings. On one hand, as mentioned earlier, bvFTD and AD are associated with different cognitive profiles, but there is significant overlap (Musa et al., 2020). On the other hand, although each cognitive function is associated with more or less specific neural systems, multiple mechanisms and functional cognitive systems underlie the impairment of particular cognitive tasks. Current models of cognitive functions have revealed a more complex and distributed neural basis than previously assumed in traditional theories of localization of cognitive abilities. In this regard, the knowledge of the neural basis of cognitive tests has practical implications in clinical practice to improve the interpretation of findings and theoretical relevance for advancing our understanding of brain-behavior relationships. However, relatively few works have explored this topic in the context of brief cognitive tests (Paul et al., 2011). For instance, some studies have examined the capacity of MoCA to predict brain metabolism in patients with mild cognitive impairment due to AD and transient ischemic attack or lacunar stroke and hippocampal atrophy in patients with memory complaints (Ritter et al., 2017; Zukotynski et al., 2020). To the best of our knowledge, there are no previous studies analyzing the neural basis of the ACE-III.

In this study, we aimed to examine the neural correlates of ACE-III in a large cohort of 300 participants with bvFTD and AD. This information may be valuable in interpreting the test and its cognitive domains. We also aimed to evaluate the diagnostic properties of ACE-III and determine the best cutoff points based on the clinical diagnosis and brain metabolism.

## Methods

### Study design and population

We enrolled 300 participants in a cross-sectional study, including 180 patients with AD, 60 with behavioral FTD, and 60 controls. Patients were diagnosed according to the current diagnostic criteria. Patients with bvFTD met the diagnostic criteria by Rascovsky et al. (2011) and had at least 2 years of follow-up confirming the diagnosis. Patients with AD were diagnosed according to the criteria by McKhann et al. (2011) and had neuroimaging and/or CSF confirmation (Aβ1-42, tau and phospho-tau) (McKhann et al., 2011; Rascovsky et al., 2011). In cases of bvFTD, CSF biomarkers were used when considered clinically necessary. Cognitive assessment were carried out using a standardized protocol encompassing the following tests: digit span forward and backward; Corsi’s cubes forward and backward; Boston Naming Test; Trail Making Test parts A and B; Symbol Digit Modalities test; Free and Cued Selective Reminding Test; Rey-Osterrieth Complex Figure (copy and memory at 3 and 3 min); verbal fluency (semantic and letter); and Visual Object and Space Perception Battery (subtests object decision, progressive silhouettes, position discrimination, and number location). Additionally, global CDR for staging of AD patients and global CDR plus NACC FTLD rating for bvFTD were used (Morris, 1993; Miyagawa et al., 2020).

Atypical variants of AD (consisting of 15 patients with posterior cortical atrophy and 104 with logogenic aphasia) and language variants of FTD (comprising 87 patients with non-fluent primary progressive aphasia and 43 with semantic aphasia) were excluded from this study. Only the initial FDG-PET imaging of each patient was used. The recruitment took place at the Department of Neurology of the Hospital Clinico San Carlos (Madrid, Spain) between February 2015 and February 2021.

### Addenbrooke’s cognitive examination III

All participants underwent ACE-III testing, which includes the following scores: total score (the sum of all items) and five domains: attention (scored between 0 and 18), memory (0–26), fluency (0–14), language (0–26), and visuospatial abilities (0–16) (Hsieh et al., 2013; Matías-Guiu et al., 2015). The attention domain comprises the following tasks: time orientation (0–5), spatial orientation (0–5), repetition of 3 words (0–3), and serial subtractions (0–3). The memory domain involves recalling of 3 previously repeated words, learning of a name and address of a person (0–7), recalling known historical and present facts (0–4), and delayed recall of the name and address (0–7). The fluency domain is assessed through letter (words beginning with “p”) (0–7) and semantic (animals) verbal fluency (0–7). The language domain encompasses the following tasks: understanding a set of physical commands (0–3); writing two complete sentences (0–2); repetition of complex words (0–2) and sentences (0–2); naming 12 drawings (0–12); semantic knowledge about the previous drawings (0–4); and reading of five stranger words to assess surface dyslexia (0–1). Finally, the visuospatial abilities domain comprises three visuoconstructive tasks (copying two loops (0–1), a cube (0–2) and drawing a clock (0–5)), one visuospatial (counting a set of dots) (0–4), and one visuoperceptive task (recognizing four incomplete letters) (0–4).

The test was administered according to the guidelines and materials provided for the Spanish-language version and can be accessed at https://frontierftd.org/.

### Acquisition, preprocessing, and analysis of FDG-PET imaging

PET-CT imaging was conducted using a Siemens Biograph True Point Platform equipped with a 6-slice detector. Patients fasted for a minimum of 6 h before receiving an average dose of ^18^F-FDG of 185 MBq. A static PET image was acquired through a sinogram bed 30 min after administering the tracer, along with the rest of the patient. CT parameters were set as follows: 130/40/1 (kVp/effective mAs/rotation time); slice thickness of 3 mm; reconstruction interval of 1.5 mm; and pitch of 0.75. Subsequently, iterative 3D image reconstruction was performed using the True X method with two iterations and 21 subsets. The interval between cognitive assessment and FDG-PET was less than 3 months for all patients.

Statistical Parametric Mapping 12 (SPM12) (The Wellcome Trust Centre for Neuroimaging, Institute of Neurology, University College of London) was used for preprocessing and analysis of FDG-PET imaging^1^. Images were first realigned and normalized to the Montreal Neurological Institute (MNI) and then smooth at 8 mm full width at half maximum. Global metabolism was introduced as a nuisance covariate.

Multiple regression analysis was used to study the positive correlation between ACE-III scores and brain metabolism at a voxel level. Age, gender, and years of education were introduced as covariates. Furthermore, a two-sample *T*-test was used to define the regions impaired in each group against controls. These analyses were performed using SPM12, with a statistical threshold of FWE-corrected (cluster level) value of *p* <0.05. SPM maps are presented in neurological orientation, with the left hemisphere on the left-side, and the right hemisphere on the right. The MNI coordinate system was used for the localization of the regions in the standard space.

### Statistical analysis

Statistical analysis was performed using the software IBM® SPSS Statistics 26.0. Descriptive results are shown as mean ± standard deviation or frequency (percentage). Normality was assessed using the Kolmogorov–Smirnov test. To assess differences among the three groups (bvFTD, AD, HC), a Kruskall-Wallis test with posthoc Dunn analysis was conducted. A value of *p* <0.05 was considered statistically significant.

Receiver Operating Characteristic (ROC) curves were calculated to evaluate the discrimination between bvFTD vs. HC, and AD vs. HC using the ACE-III. Additionally, discrimination between normal and altered metabolism in the key regions associated with bvFTD and AD, as depicted in Supplementary Figure S1, was evaluated. An area under the curve (AUC) >0.7 was deemed acceptable. Youden’s index (YI) was calculated to determine the best cutoff points, and various diagnostic metrics, including sensitivity, specificity, positive and negative predictive values, positive and negative likelihood values, and Number Needed for Screening Utility (NNSU) were also computed (Larner, 2019).

## Results

### Ace-III performance across groups

Patients with bvFTD showed lower performance than controls in all ACE-III domains and in the total score. Similarly, patients with AD scored lower than controls in all the domains. Finally, patients with bvFTD scored lower than AD in fluency and language domain (Table 1 and Supplementary Table S1). The AUC for the discrimination between bvFTD and HC was 0.871 using the total score (*p* < 0.001). The best cutoff was 81 (YI = 0.683, Sensitivity 85%, Specificity 83.3%). The AUC for distinguishing AD and HC was 0.834 (*p* < 0.001). In this case, the best cutoff was 85 (YI = 0.528, Sensitivity 82.8%, Specificity 70%). All the metrics evaluating test accuracy are shown in Table 2. AUC and cutoff scores for each ACE-III domain are shown in Supplementary Table S2.

**Table 1:** Main demographic characteristics and ACE-III performance across groups.

		bvFTD (*n* = 60)	AD (*n* = 180)	HC (*n* = 60)	H /X^2^ (value of *p*)
Age		71.13 ± 7.97	72.84 ± 6.33	71.03 ± 5.59	6.23 (0.044)
Sex (women)		24 (40.0%)	97 (53.9%)	36 (60%)	5.24 (0.073)
Years of education		10.12 ± 4.49	10.11 ± 5.00	11.40 ± 4.14	3.88 (0.143)
CDR global^*^	0.5	17 (28.3%)	101 (56.1%)	–	21.36 (<0.001)
	1	28 (46.7%)	64 (35.6%)		
	2	13 (21.7%)	15 (53.6%)		
	3	2 (3.3%)	0 (0%)		
ACE-III (total score)^a,b^		64.80 ± 18.01	69.52 ± 16.97	87.13 ± 8.22	70.62 (<0.001)
ACE-III (attention)^a,b^		13.88 ± 3.67	13.96 ± 3.39	16.83 ± 1.17	45.02 (<0.001)
ACE-III (memory)^a,b^		13.80 ± 5.88	13.68 ± 5.67	20.57 ± 4.23	61.17 (<0.001)
ACE-III (fluency)^a,b,c^		5.75 ± 3.78	8.23 ± 3.55	11.08 ± 1.89	61.32 (<0.001)
ACE-III (language)^a,b,c^		18.88 ± 4.95	21.04 ± 4.69	23.90 ± 2.66	40.89 (<0.001)
ACE-III (visuospatial)^a,b^		12.08 ± 3.04	12.49 ± 2.99	14.70 ± 1.49	35.82 (<0.001)

Kruskall-Wallis with *post-hoc* analysis (adjusted by Bonferroni) showed statistically significant differences after Bonferroni correction between bvFTD vs HC (a), AD vs HC (b), and bvFTD vs AD (c).

^*^CDR global for AD and global CDR plus NACC FTLD for bvFTD.

**Table 2:** Metrics for test accuracy using ACE-III (total score).

	Diagnosis		Hypometabolism in FDG-PET	
	AD vs. HC	bvFTD vs. HC	AD-regions	bvFTD-regions
AUC	0.834	0.871	0.704	0.805
Best cutoff	85	81	84	80
Sensitivity	82.8%	85.0%	91.1%	78.9%
Specificity	70%	83.3%	40.2%	77.7%
PPV	89.2%	83.6%	20.1%	76.2%
NPV	57.53%	84.7%	96.5%	80.3%
LR+	2.76	5.08	1.53	3.53
LR-	0.24	0.18	0.22	0.27
NNSU	0.87	0.70	1.75	0.81

AUC, area under the curve; PPV, positive predictive value; NPV, negative predictive value; LR+, positive likelihood ration; LR-, negative likelihood ratio; NNSU, Number Needed for Screening Utility.

### Metabolic correlates of ACE-III in bvFTD

The total score was positively correlated with the metabolism of the left superior, middle, and superior medial frontal gyrus.

The attention domain was correlated with the left superior and middle frontal gyri, supplementary motor area, precentral gyrus and cingulate gyrus (anterior and mid parts). The memory domain was associated with the metabolism of the left insula, inferior, middle and superior frontal gyri, superior temporal gyrus, and anterior cingulate.

The fluency domain was correlated with two clusters, including the left frontal lobe (superior, middle, and inferior frontal gyri, precentral, middle and anterior cingulate) and extending to the left temporal (superior, middle and inferior temporal gyri), parietal (left inferior parietal lobule, angular and supramarginal gyri) and insula.

The language domain was not associated with any significant cluster at the prespecified threshold.

**Figure 1:**
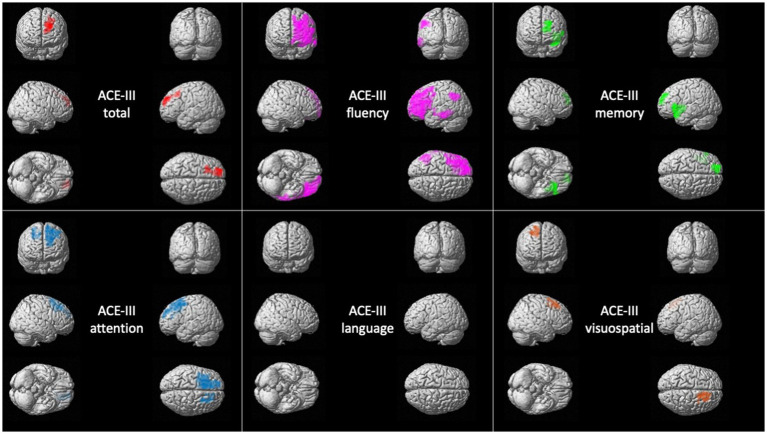
Voxel-based brain mapping analysis showing the correlation between ACE-III scores and brain metabolism in bvFTD (FWE-corrected value of *p* <0.05). ACE-III total score is represented in *red,* ACE-III attention in *blue*, ACE-III fluency in *violet*, ACE-III language in *yellow*, ACE-III memory in *green*, and ACE-III visuospatial in *orange*.

The visuospatial domain was correlated with the metabolism of the right superior and middle frontal gyri and the supplementary motor area (Figure 1).

Complete details about statistics are shown in Supplementary Table S3.

### Metabolic correlates of ACE-III in AD

The total score was correlated with the metabolism of three large clusters involving the bilateral temporoparietal lobes and extending to some occipital regions.

The attention domain was correlated with bilateral superior, middle, and inferior temporal gyri, posterior cingulate, precuneus, inferior parietal lobule and bilateral angular and fusiform gyri.

The memory domain was correlated with bilateral superior, middle, and inferior temporal gyri, left parahippocampal gyrus and hippocampus, posterior and middle cingulate gyri, precuneus, and inferior parietal lobule.

The fluency domain was associated with a large cluster in the left hemisphere involving the superior, middle, and inferior temporal gyri, inferior and middle frontal gyri, angular and fusiform gyri, precuneus, anterior, superior and inferior parietal lobule, middle and posterior cingulate. It was also associated with a smaller cluster in the right temporal lobe.

The language domain was associated with the metabolism of the left hemisphere, especially with temporoparietal regions and left inferior and middle frontal gyri. It was also correlated with the right temporal lobe.

**Figure 2:**
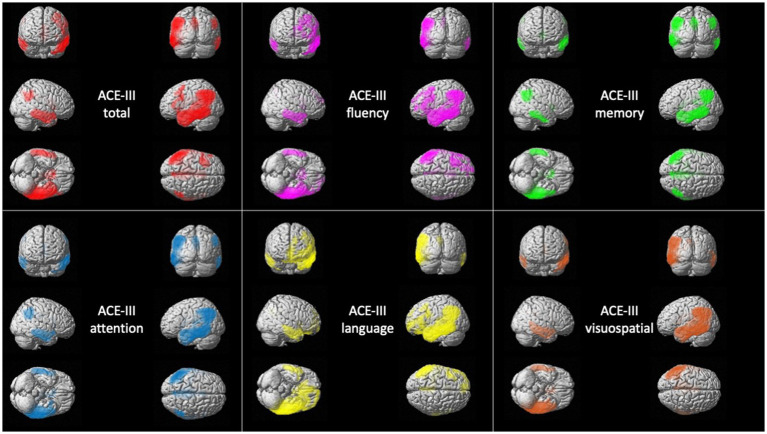
Voxel-based brain mapping analysis showing the correlation between ACE-III scores and brain metabolism in AD (FWE-corrected value of *p* <0.05). ACE-III total score is represented in *red,* ACE-III attention in *blue*, ACE-III fluency in *violet*, ACE-III language in *yellow*, ACE-III memory in *green*, and ACE-III visuospatial in *orange*.

The visuospatial domain was correlated with bilateral temporal lobe, and left inferior parietal lobule, angular and supramarginal gyri, precuneus, posterior cingulate, lingual gyrus, and middle occipital gyrus (Figure 2).

Complete details about statistics are shown in Supplementary Table S4.

### BvFTD and AD metabolism and staging according to ACE-III

**Figure 3:**
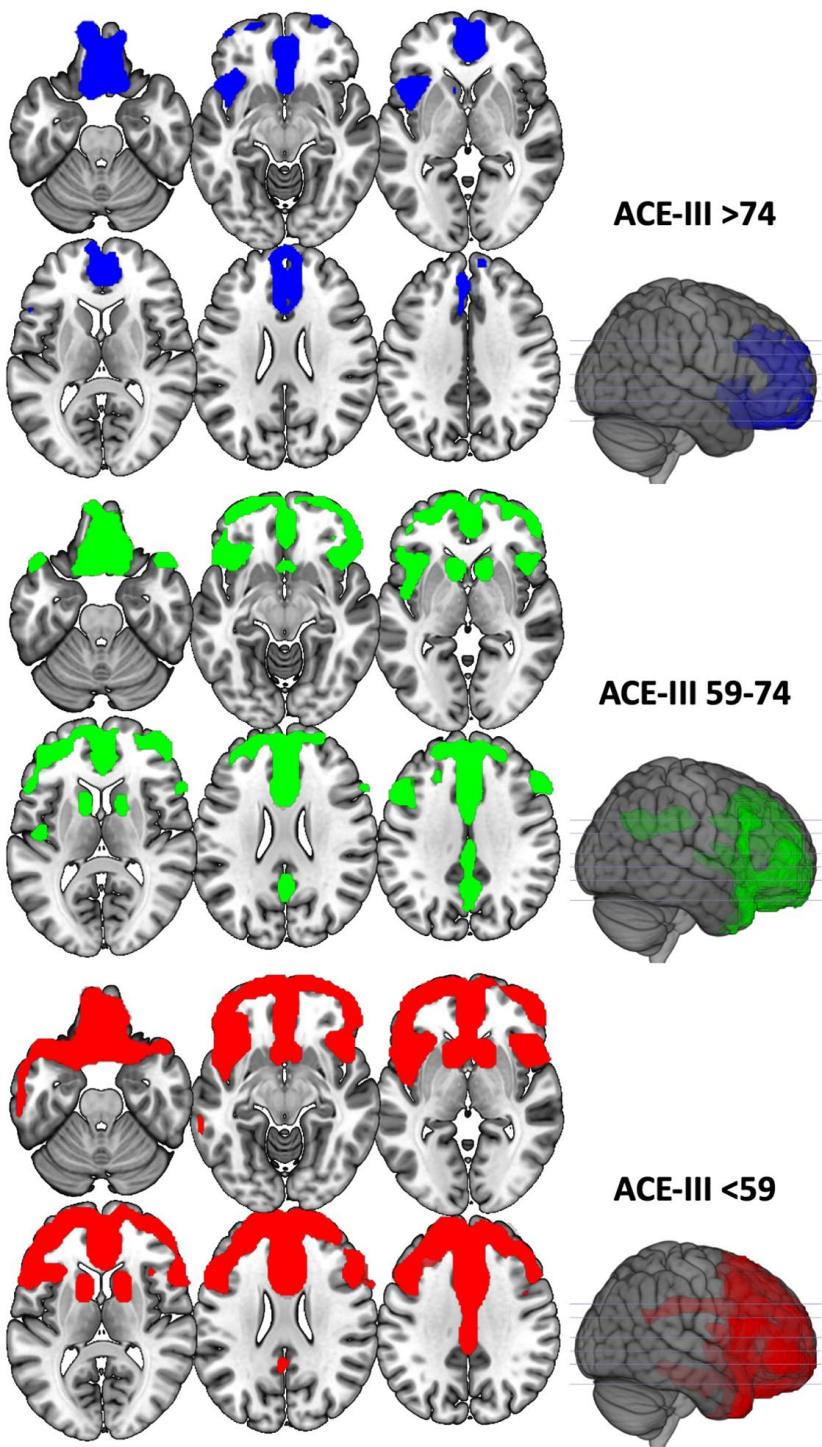
Voxel-based brain mapping analysis showing the regions with a lower brain metabolism in each of the tertile groups of bvFTD against HC (FWE-corrected value of *p* <0.05). ACE-III scores of each group are shown.

**Figure 4:**
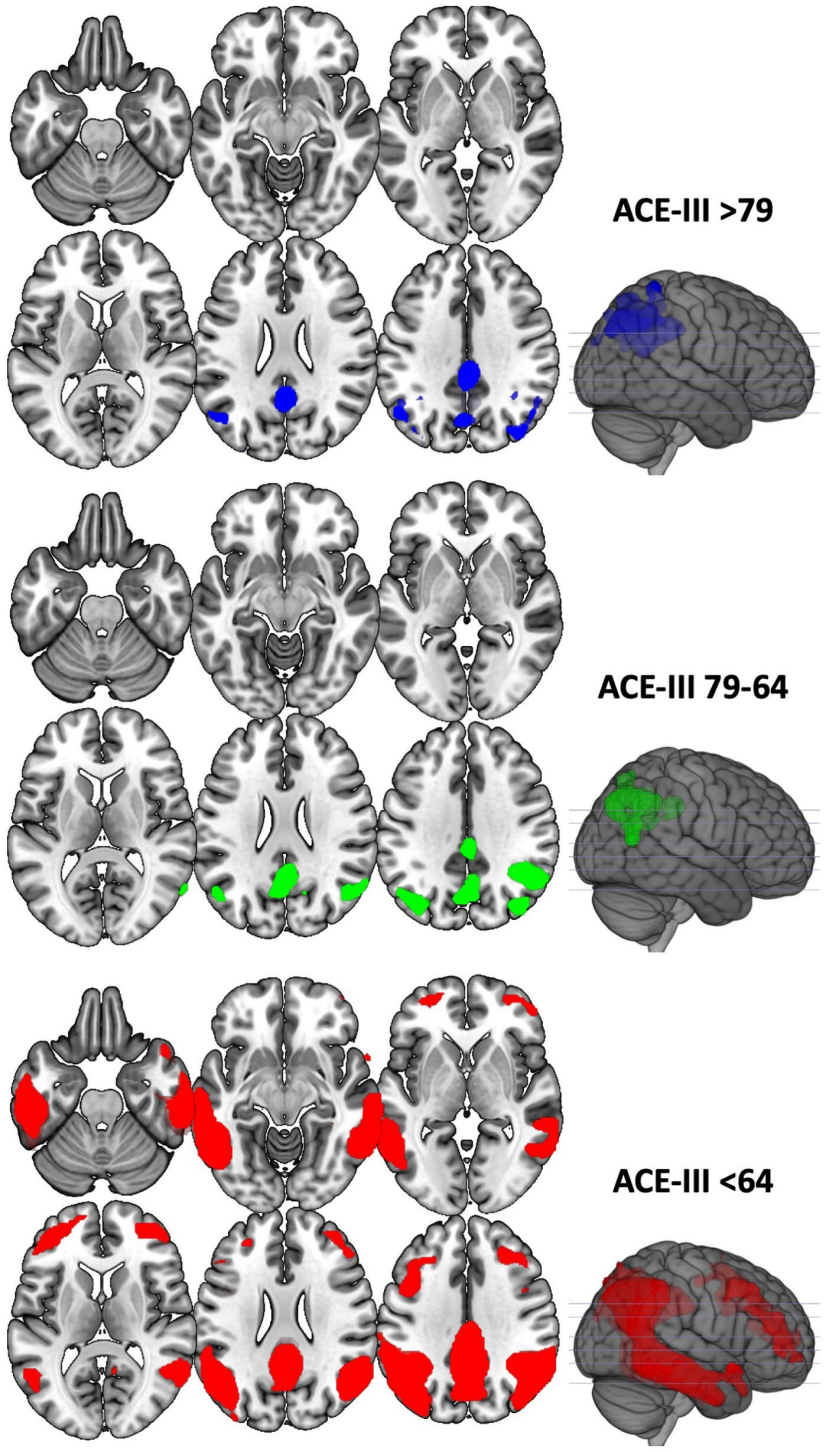
Voxel-based brain mapping analysis showing the regions with a lower brain metabolism in each of the tertile groups of AD against HC (FWE-corrected value of *p* <0.05). ACE-III scores of each group are shown.

Patients with bvFTD and AD were divided into three tertiles according to the ACE-III total score, and each group was compared with controls (Figures 3, 4). Complete statistics are detailed in Supplementary Table S5.

### Estimation of cutoff scores based on FDG-PET imaging

The AUC for the detection of hypometabolism in FTD-regions using the ACE-III (total score) was 0.805. The best cutoff was 80 (YI = 0.567, sensitivity 78.95%, specificity 77.78%). The AUC for the detection of hypometabolism in AD-regions was 0.704. The best cutoff was 84 (YI = 0.315, sensitivity 91.18%, specificity 40.29%). All the metrics evaluating test accuracy are shown in Table 2 for ACE-III total score and Supplementary Table S2 for domains scores.

## Discussion

In this study, we aimed to evaluate the neural correlates of the ACE-III and its domains. We explored the relationship between each ACE-III score and resting-state brain metabolism in two patient cohorts: those with bvFTD and AD. As expected, all ACE-III scores were significantly lower in these patient groups compared with HC, reflecting the global cognitive impairment that occurs as these neurodegenerative diseases progress.

In the comparison between AD and bvFTD, verbal fluency and language scores were lower in bvFTD, which is consistent with other studies (Siri et al., 2001). However, these findings are not replicated in all the FTD cohorts, due to the heterogeneity of these disorders, particularly FTD, and the time of assessment throughout the disease (1; (Siri et al., 2001; Reul et al., 2017; Bruno et al., 2020)). Verbal fluency in the ACE-III encompasses both semantic and letter fluency tasks, which draw on multiple cognitive domains, including memory and executive function. Therefore, it is impaired in both AD and bvFTD. However, individuals with more severe executive dysfunction may exhibit more pronounced deficits in both semantic and letter fluency, while patients with prominent episodic memory deficits generally impair only semantic fluency. In terms of the language domain, our study showed that it was more impaired in bvFTD than AD. The language domain involves naming abilities (12 out of 26 points), but also includes semantic tasks (5 points) or writing (2 points), among others. These tasks likely engage both language and executive networks, reflecting the heterogeneous yet significant language deficits previously described in other bvFTD cohorts (Hardy et al., 2016; Geraudie et al., 2021). Despite these differences, it is important to recognize that there is a substantial overlap in scores between AD and bvFTD groups (as in other cognitive tests), and no single score alone is sensitive enough to reliably differentiate between these disorders. Additionally, bvFTD were more functionally impaired than AD patients.

Furthermore, our analysis revealed that the total ACE-III score was correlated with brain regions typically impaired in the early stages of bvFTD and AD. Notably, there was a certain left hemisphere predominance in both cases. This may be attributed with the fact that the majority ACE-III items involve verbal input or output. This would suggest that the test could be less sensitive to cases with predominant right hemisphere damage, which could be especially important in some variants of FTD in which asymmetry is more frequent than in AD. However, this left hemisphere bias could also be explained by the left hemisphere’s higher susceptibility to neurodegeneration in these disorders (Thompson et al., 2003; Shi et al., 2009; Rohrer, 2012; Donix et al., 2013; Whitwell et al., 2013).

Interestingly, the ACE-III domains exhibited different neural correlates in bvFTD. Almost all the scores showed a left hemisphere predominance. Memory was associated with left frontal lobe function, and with a smaller cluster in the left temporal lobe. This confirms that frontal lobe dysfunction can produce memory impairment in bvFTD, supporting recent evidence that emphasizes episodic memory dysfunction in at least a subgroup of patients with bvFTD (Fernandez-Matarrubia et al., 2017; Poos et al., 2018). Similarly, verbal fluency was more strongly associated with the left frontal lobe, extending to left temporal and parietal regions, according to previous literature (Delgado-Álvarez et al., 2022). Interestingly, the visuospatial domain was correlated with the right superior and middle frontal gyri and supplementary motor area. This may be interpreted considering the role of prefrontal and premotor regions in visuospatial processing and the role of the right frontal cortex in planning of visuoconstructive tasks (Delgado-Álvarez et al., 2022). The different neural correlates of the ACE-III domains could also be indirect evidence of the heterogeneity of this disorder, in which different cognitive profiles may be found.

We did not detect statistically significant associations with the language domain in the bvFTD. This could be explained because patients with language variants of FTD were specifically excluded. In this regard, the comparison between bvFTD and HC showed a relative sparing of brain regions more closely associated with language dysfunction in FTD (i.e., perisylvian regions and left anterior temporal lobe). Even after excluding patients with PPA, individuals with bvFTD show a range of mild language disorders, including semantic processing, comprehension skills, naming difficulties (Geraudie et al., 2021). This heterogeneity may explain the lack of clear correlations with the ACE-III language domain.

Regarding AD, the different ACE-III domains were correlated with the main regions affected in the early stages of AD, comprising the bilateral temporoparietal lobes. These brain correlates encompassed some of the earliest regions involved in AD, such as the precuneus, posterior cingulate, and middle temporal gyrus. Interestingly, the attention domain was more closely associated with parietal and temporal regions, rather than frontal regions. This alignment is consistent with the inclusion of orientation and calculation tasks within this domain, as well as the known association associative brain regions and attention and executive function in AD (Habeck et al., 2012; Matías-Guiu et al., 2017). In the visuospatial domain, the correlated regions also extended to the occipital lobe.

Overall, our findings support the notion of a distinct neural basis for ACE-III in bvFTD and AD. This highlights that specific cognitive scores within ACE-III may reflect dysfunction in various and heterogeneous brain regions. For instance, memory domain impairment may be associated with the left frontal lobe in cases with bvFTD or bilateral parieto-temporal lobe in AD. This should be considered when interpreting the results from each cognitive domain, in which the information of each domain should be put into the context of the other cognitive domains, behavior changes and functional status.

Another interesting result of our study is the demonstration that ACE-III is sensitive to changes in brain metabolism. This suggests that ACE-III can be used for monitoring and staging patients during their follow-up. In this regard, upper tertiles of the test were associated with the earliest regions impaired in bvFTD and AD, whereas lower tertiles were linked to more advanced stages of each disorder. These findings are consistent with previous studies that have correlated ACE-III scores with functional abilities (So et al., 2018), underscoring ACE-III as a reliable tool for patient follow-up and monitoring.

In this study, we also proposed some ACE-III cutoffs based on brain metabolism. The determination of the best cutoffs for neuropsychological tests is a controversial issue. The most used procedure is the estimation of ROC curves by comparing a diagnosis with a control group, and the calculation of a cutoff point according to the levels of sensitivity and specificity. However, fixed cutoffs may be less useful in some settings (e.g., lower levels of schooling) or disorders with a wide range of ages in the presentation. In this case, the collection of normative data may be a solution, and in the case of ACE-III it improved the diagnosis (Matías-Guiu et al., 2016). However, in this case, the choice of the specific cutoff point (e.g., 1, 1.5 or 2 standard deviations below the norms) remains challenging. In this study, we examined the use of cutoff points based on biomarkers of brain function. In this regard, the good AUC values for detecting hypometabolism in bvFTD and AD regions support the utility of ACE-III, complementing information from normative data and prior validation studies. It is worth noting that the AUC for detecting hypometabolism in the earliest regions impaired in AD was lower than in the regions impaired in bvFTD. Specifically, specificity and positive predictive values were limited when diagnosing AD versus HC. Utilizing more challenging and specific memory paradigms may offer improved diagnostic capacity in this context (Curiel-Cid et al., 2022; Valles-Salgado et al., 2022). In addition, patients with AD generally seek medical attention earlier than those with bvFTD (Ellajosyula et al., 2022), and in our cohort, patients with AD were diagnosed at earlier stages according to CDR. Similarly, combining ACE-III with other tests examining cognitive functions early impaired in bvFTD (e.g., social cognition) could improve the diagnostic capacity (Dodich et al., 2018; García-Gutiérrez et al., 2021). However, due to the difficulties in detecting early stages of AD and bvFTD, the AUC values based on brain metabolism are appropriate considering the time of administration of the test for a first assessment of patients. In our study, FDG-PET calculated cutoffs are consistent with those estimated by comparing patients with controls and are comparable to those proposed by other researchers in various cohorts using clinical criteria (Hsieh et al., 2013; Reul et al., 2017). This underscores the consistency of ACE-III in detecting AD and bvFTD and suggests a potential cross-cultural equivalence. Based on these findings, ACE-III should be regarded as a valuable screening tool, given its favorable sensitivity, but findings should be confirmed through additional cognitive tests or biomarkers due to its relatively low specificity, especially in the context of AD symptoms.

Several limitations of our study should be acknowledged. Firstly, we focused our analysis on the total ACE-III score and domains scores. Exploring individual items might enhance the differentiation between disorders, as demonstrated in the case of primary progressive aphasia variants (Foxe et al., 2022). In addition, qualitative assessment of individual tasks could also provide valuable insights for diagnosis, as differences in error patterns between bvFTD and AD may exist (Musa et al., 2020). Secondly, due to the progressive nature of neurodegenerative disorders and the involvement of several cognitive domains concurrently isolating cognitive functions for the analysis of their neural correlates is difficult. Due to the high correlation between the different domain scores, we could not control for the other scores to prevent the effect of collinearity. Thirdly, we have restricted our analysis to the most prototypical variants of AD (amnestic type) and FTD (behavioral variant). Future studies should aim to confirm these findings in the atypical variants of AD and FTD are necessary. Fourthly, pathological confirmation of the diagnosis was not available, and amyloid biomarkers were not available in all cases. However, the group comparison of FDG-PET imaging against controls confirmed the expected brain regions impaired in each disease.

In conclusion, our study contributes to the knowledge of the brain regions associated with ACE-III, improving the interpretation of the test and suggesting the usefulness of this test for screening and monitoring. These findings provide further evidence of the validity of ACE-III for assessing patients with bvFTD and AD in both clinical and research settings.

## Data availability statement

The raw data supporting the conclusions of this article will be made available by the authors, without undue reservation.

## Ethics statement

The studies involving humans were approved by Comité de Ética de Hospital Clinico San Carlos. The studies were conducted in accordance with the local legislation and institutional requirements. The participants provided their written informed consent to participate in this study.

## Author contributions

MC-M: Conceptualization, Data curation, Formal analysis, Funding acquisition, Investigation, Writing – original draft, Writing – review & editing. PN: Data curation, Investigation, Writing – review & editing. MV-S: Data curation, Investigation, Writing – review & editing. PB: Investigation, Writing – review & editing. CD-A: Data curation, Investigation, Writing – review & editing. AD-A: Data curation, Investigation, Writing – review & editing. LF-R: Data curation, Investigation, Writing – review & editing. JL-C: Data curation, Investigation, Writing – review & editing. MD-C: Investigation, Writing – review & editing. MG-M: Investigation, Writing – review & editing. JM-G: Funding acquisition, Investigation, Supervision, Visualization, Writing – review & editing. JAM-G: Conceptualization, Data curation, Formal analysis, Funding acquisition, Investigation, Methodology, Supervision, Visualization, Writing – original draft, Writing – review & editing.

## Footnotes

^1^ https://www.fil.ion.ucl.ac.uk/spm/
