# Supplementary material for: FDG-PET-based neural correlates of Addenbrooke’s cognitive examination III scores in Alzheimer’s disease and frontotemporal degeneration
Source: Front Psychol. 2023 Nov 16;14:1273608. doi: 10.3389/fpsyg.2023.1273608 (PMC10687370; doi:10.3389/fpsyg.2023.1273608)
Supplement: Supplementary file 1 [file Table_1.DOCX]

**Supplementary Table 1**

| Post-hoc analysis to the Table 1. Comparison of demographic characteristics and ACE-III performance across groups. P-values (adjusted by Bonferroni) are shown. | | | |
| --- | --- | --- | --- |
|  | **bvFTD vs HC** | **AD vs HC** | **bvFTD vs AD** |
| Age | 1.00 | 0.057 | 0.465 |
| Years of education | - | - | - |
| ACE-III (total score)^a,b^ |  |  | 0.241 |
| ACE-III (attention)^a,b^ | <0.001 | <0.001 | 1.00 |
| ACE-III (memory)^a,b^ | <0.001 | <0.001 | 1.00 |
| ACE-III (fluency)^a,b,c^ | <0.001 | <0.001 | <0.001 |
| ACE-III (language)^a,b,c^ | <0.001 | <0.001 | 0.005 |
| ACE-III (visuospatial)^a,b^ | <0.001 | <0.001 | 0.986 |
