# Supplementary material for: FDG-PET-based neural correlates of Addenbrooke’s cognitive examination III scores in Alzheimer’s disease and frontotemporal degeneration
Source: Front Psychol. 2023 Nov 16;14:1273608. doi: 10.3389/fpsyg.2023.1273608 (PMC10687370; doi:10.3389/fpsyg.2023.1273608)
Supplement: Supplementary file 2 [file Table_2.DOCX]

| **Supplementary Table 2**. Metrics for test accuracy using ACE-III (domain scores) | | | | |
| --- | --- | --- | --- | --- |
|  | Diagnosis | | Hypometabolism in FDG-PET | |
|  | AD vs HC | bvFTD vs HC | AD-regions | bvFTD-regions |
| *ACE-III attention* | | | | |
| AUC | 0.779 | 0.772 | 0.679 | 0.718 |
| Best cutoff | 15 | 15 | 12 | 15 |
| Sensitivity | 58.8% | 61.6% | 47.0% | 57.8% |
| Specificity | 86.6% | 86.67% | 82.5% | 80.95% |
| *ACE-III memory* | | | | |
| AUC | 0.829 | 0.818 | 0.698 | 0.792 |
| Best cutoff | 16 | 16 | 14 | 16 |
| Sensitivity | 71.6% | 71.6% | 50% | 71.9% |
| Specificity | 86.6% | 86.6% | 75.7% | 84.1% |
| *ACE-III fluency* | | | | |
| AUC | 0.741 | 0.885 | 0.645 | 0.808 |
| Best cutoff | 8 | 9 | 6 | 7 |
| Sensitivity | 51.1% | 85% | 50% | 63.1% |
| Specificity | 90% | 78.3% | 79.1% | 88.8% |
| *ACE-III language* | | | | |
| AUC | 0.701 | 0.821 | 0.643 | 0.735 |
| Best cutoff | 23 | 23 | 23 | 23 |
| Sensitivity | 61.1% | 78.3% | 73.5% | 73.6% |
| Specificity | 73.3% | 73.3% | 50.9% | 66.6% |
| *ACE-III visuospatial* | | | | |
| AUC | 0.733 | 0.776 | 0.627 | 0.733 |
| Best cutoff | 13 | 14 | 11 | 14 |
| Sensitivity | 56.6% | 73.33% | 44.1% | 71.9% |
| Specificity | 80% | 65% | 76.7% | 61.9% |
