# Supplementary material for: FDG-PET-based neural correlates of Addenbrooke’s cognitive examination III scores in Alzheimer’s disease and frontotemporal degeneration
Source: Front Psychol. 2023 Nov 16;14:1273608. doi: 10.3389/fpsyg.2023.1273608 (PMC10687370; doi:10.3389/fpsyg.2023.1273608)
Supplement: Supplementary file 3 [file Table_3.DOCX]

| **Supplementary Table 3.** Voxel-based brain mapping analysis results in **bvFTD.**  Correlation with neuropsychological tests, using an uncorrected p-value <0.001 and a FWE-cluster based corrected p-value <0.05 | | | | | | |
| --- | --- | --- | --- | --- | --- | --- |
| Brain regions  (localization of peak coordinates are shown in **bold**) | MNI coordinates | | | T value | Z score | K (number of voxels) |
|  | x | y | z |  |  |  |
| *Regions correlated with* ***ACE-III (total)*** | | | | | | |
| **Left superior** **frontal**, middle frontal and **superior medial fronta**l gyri. | -22 | 52 | 34 | 4.95 | 4.47 | 1469 |
|  | -22 | 24 | 44 | 4.15 | 3.85 |  |
|  | -8 | 56 | 16 | 3.76 | 3.53 |  |
| *Regions correlated with* ***ACE-III (attention)*** | | | | | | |
| **Left superior frontal** and middle frontal gyri; supplementary motor area, precental, and cingulate gyrus (mid and anterior parts). | -26 | 0 | 56 | 5.30 | 4.73 | 4907 |
|  | -14 | 14 | 66 | 4.84 | 4.39 |  |
|  | -22 | 24 | 48 | 4.79 | 4.36 |  |
| **Left superior and middle frontal gyri;** supplementary motor area. | 24 | 36 | 44 | 4.42 | 4.06 | 988 |
|  | 30 | 20 | 38 | 3.91 | 3.65 |  |
|  | 12 | 22 | 52 | 3.88 | 3.63 |  |
| *Regions correlated with* ***ACE-III (memory)*** | | | | | | |
| Left insula, inferior frontal and **superior temporal** gyri. **Left insula.** | -40 | 6 | 2 | 4.72 | 4.30 | 2232 |
|  | -36 | 20 | 4 | 4.23 | 3.91 |  |
|  | -42 | 20 | -20 | 4.18 | 3.88 |  |
| **Left superior frontal** and middle frontal, superior **medial frontal** and anterior cingulate | -20 | 50 | 34 | 4.59 | 4.20 | 1056 |
|  | -20 | 58 | 26 | 4.38 | 4.03 |  |
|  | -6 | 56 | 14 | 4.33 | 4.00 |  |
| *Regions correlated with* ***ACE-III (fluency)*** | | | | | | |
| **Left superior, middle and inferior frontal gyri**; left middle and inferior temporal gyri; precentral, insula, middle and anterior cingulate. | -22 | 52 | 32 | 6.70 | 5.69 | 14577 |
|  | -50 | 34 | -8 | 5.58 | 4.93 |  |
|  | -24 | 24 | 40 | 5.37 | 4.78 |  |
| **Left inferior parietal lobule, angular**, supramarginal and **superior temporal gyri**. | -42 | -54 | 38 | 5.04 | 4.54 | 1288 |
|  | -48 | -72 | 38 | 4.16 | 3.86 |  |
|  | -58 | -62 | 28 | 3.80 | 3.56 |  |
| *Regions correlated with* ***ACE-III (language)*** | | | | | | |
| Not suprathreshold clusters | | | | | | |
|  |  |  |  |  |  |  |
|  |  |  |  |  |  |  |
| Regions correlated with ***ACE-III (visuospatial)*** | | | | | | |
| **Right superior frontal** and middle frontal, supplementary motor area. | 18 | 8 | 66 | 4.69 | 4.27 | 1361 |
|  | 12 | 20 | 52 | 4.63 | 4.23 |  |
|  | 26 | 30 | 50 | 4.59 | 4.20 |  |
