# Supplementary material for: FDG-PET-based neural correlates of Addenbrooke’s cognitive examination III scores in Alzheimer’s disease and frontotemporal degeneration
Source: Front Psychol. 2023 Nov 16;14:1273608. doi: 10.3389/fpsyg.2023.1273608 (PMC10687370; doi:10.3389/fpsyg.2023.1273608)
Supplement: Supplementary file 4 [file Table_4.DOCX]

| **Supplementary Table 4.** Voxel-based brain mapping analysis results in **AD.**  Correlation with neuropsychological tests, using an uncorrected p-value <0.001 and a FWE-cluster based corrected p-value <0.05  (*) means “negative correlation”; in other tests, positive correlations are shown. | | | | | | |
| --- | --- | --- | --- | --- | --- | --- |
| Brain regions  (localization of peak coordinates are shown in **bold**) | MNI coordinates | | | T value | Z score | K (number of voxels) |
|  | x | y | z |  |  |  |
| *Regions correlated with* ***ACE-III (total)*** | | | | | | |
| **Left** superior, **middle** and inferior **temporal** gyri; left inferior parietal lobule; left angular, supramarginal and **fusiform** gyri; bilateral precuneus, left middle occipital, cuneus and lingual gyri; bilateral middle and posterior cingulate gyrus; left parahippocampal and hippocampus. | -64 | -34 | -8 | 10.21 | Inf. | 28733 |
|  | -58 | -20 | -22 | 9.85 | Inf. |  |
|  | -50 | -60 | 24 | 9.72 | Inf. |  |
| **Right** superior, **middle** and inferior **temporal** gyri, and **fusiform** gyrus. | 62 | -20 | -26 | 5.47 | 5.24 | 2940 |
|  | 64 | -22 | -10 | 5.04 | 4.86 |  |
|  | 56 | 2 | -24 | 4.82 | 4.67 |  |
| **Right** angular and **supramarginal gyri; inferior parietal lobule**. | 52 | -50 | 34 | 4.47 | 4.34 | 948 |
|  | 56 | -46 | 48 | 3.85 | 3.76 |  |
| *Regions correlated with* ***ACE-III (attention)*** | | | | | | |
| **Left** superior, **middle** and inferior **temporal** gyri; left angular and fusiform gyri; left precuneus; bilateral posterior and middle cingulate; | -46 | -64 | 26 | 8.14 | 7.49 | 18068 |
|  | -64 | -36 | -10 | 7.93 | 7.32 |  |
|  | -58 | -20 | -20 | 7.76 | 7.18 |  |
| **Right** superior, **middle and inferior temporal** gyri | 62 | -22 | -24 | 5.16 | 4.97 | 2075 |
|  | 56 | 0 | -32 | 4.35 | 4.24 |  |
|  | 60 | -22 | -10 | 4.15 | 4.05 |  |
| **Right** angular and **supramarginal gyri; inferior parietal lobule.** | 54 | -52 | 30 | 4.58 | 4.44 | 1076 |
|  | 54 | -48 | 50 | 4.39 | 4.27 |  |
| *Regions correlated with* ***ACE-III (memory)*** | | | | | | |
| **Left** superior, **middle** and inferior **temporal** gyri; left angular and **fusiform**; bilateral precuneus, inferior parietal lobule; left middle occipital gyrus; bilateral posterior and middle cingulate; left parahippocampal gyrus and hippocampus; | -62 | -36 | -10 | 7.09 | 6.64 | 15196 |
|  | -50 | -60 | 26 | 6.90 | 6.47 |  |
|  | -56 | -20 | -24 | 6.50 | 6.15 |  |
| **Right** middle and **inferior temporal gyri,** and **fusiform gyrus.** | 62 | -20 | -26 | 5.22 | 5.02 | 1405 |
|  | 62 | -40 | -16 | 4.88 | 4.72 |  |
| **Right** angular and supramarginal gyri; right **inferior parietal lobule**; right **superior a**nd middle **temporal** gyri. | 50 | -56 | 26 | 4.51 | 4.38 | 1556 |
|  | 52 | -62 | 36 | 4.49 | 4.36 |  |
|  | 54 | -48 | 48 | 4.11 | 4.01 |  |
| *Regions correlated with* ***ACE-III (fluency)*** | | | | | | |
| **Left** superior, **middle**, and **inferior temporal** gyri; left inferior and middle frontal gyri; left angular and fusiform gyri; precuneus, anterior, superior and inferior parietal lobule; middle and posterior cingulate. | -60 | -20 | -20 | 9.00 | Inf. | 23998 |
|  | -60 | -40 | -16 | 8.55 | 7.79 |  |
|  | -58 | -50 | -6 | 8.06 | 7.42 |  |
| **Right** superior, **middle** and **inferior temporal** gyri. | 56 | 4 | -24 | 4.68 | 4.53 | 2203 |
|  | 64 | -18 | -14 | 4.43 | 4.31 |  |
|  | 62 | -32 | -22 | 4.06 | 3.96 |  |
| *Regions correlated with* ***ACE-III (language)*** | | | | | | |
| **Left** superior, **middle** and inferior **temporal** gyri; left supramarginal gyrus; **left fusiform;** left inferior and medial frontal gyri; left anterior and posterior cingulate | -64 | -34 | -8 | 9.51 | Inf. | 29890 |
|  | -58 | -12 | -24 | 8.75 | Inf. |  |
|  | -56 | -20 | -26 | 8.65 | Inf. |  |
| **Right** **superior, middle**, and inferior **temporal** gyrus | 62 | -22 | -10 | 5.12 | 4.94 | 2184 |
|  | 54 | 4 | -28 | 4.93 | 4.76 |  |
|  | 52 | 16 | -24 | 4.30 | 4.19 |  |
| Regions correlated with ***ACE-III (visuospatial)*** | | | | | | |
| **Left superior, middle** and inferior **temporal** gyri, parahippocampal gyrus, inferior parietal lobule, angular, **fusiform** and supramarginal gyri, precuneus, posterior cingulate, middle occipital gyrus, lingual gyrus. | -54 | -46 | 0 | 8.51 | 7.76 | 20570 |
|  | -50 | -58 | 20 | 7.83 | 7.23 |  |
|  | -52 | -44 | -22 | 7.56 | 7.02 |  |
| **Right middle** and **inferior temporal** gyri; **fusiform** gyrus. | 64 | -24 | -10 | 4.77 | 4.62 | 2185 |
|  | 58 | -6 | -18 | 4.11 | 4.01 |  |
|  | 60 | -22 | -28 | 4.05 | 3.95 |  |
