# Supplementary material for: FDG-PET-based neural correlates of Addenbrooke’s cognitive examination III scores in Alzheimer’s disease and frontotemporal degeneration
Source: Front Psychol. 2023 Nov 16;14:1273608. doi: 10.3389/fpsyg.2023.1273608 (PMC10687370; doi:10.3389/fpsyg.2023.1273608)
Supplement: Supplementary file 5 [file Table_5.DOCX]

| **Supplementary Table 5.** Voxel-based brain mapping analysis results.  Comparison between the different groups of bvFTD or AD according to the ACE-III staging (tertiles) versus healthy controls. | | | | | | |
| --- | --- | --- | --- | --- | --- | --- |
| Brain regions  (localization of peak coordinates are shown in **bold**) | MNI coordinates | | | T value | Z score | K (number of voxels) |
|  | x | y | z |  |  |  |
| ***BEHAVIORAL VARIANT FRONTOTEMPORAL DEMENTIA*** | | | | | | |
| *bvFTD (tertile 1) vs HC* | | | | | | |
| **Left** and right superior, middle and **inferior frontal** gyri; orbital frontal gyri; anterior cingulate; **insula**; **rectus** and olfactory gyri; precentral and supplementary motor area; left superior temporal gyrus and temporal pole; middle and posterior cingulum; bilateral caudate, putamen and pallidum. | -8 | 20 | -24 | 7.77 | 6.62 | 429680 |
|  | -32 | 20 | -10 | 7.66 | 6.55 |  |
|  | -40 | 10 | -2 | 7.63 | 6.53 |  |
| **Bilateral thalamus** | 8 | -18 | 6 | 5.39 | 4.94 | 654 |
|  | -4 | -18 | 8 | 5.18 | 4.77 |  |
| *bvFTD bvFTD (tertile 2) vs HC* | | | | | | |
| **Bilateral** superior, middle, and **inferior frontal** gyri; anterior cingulate and **medial frontal**; orbital, **rectal** and olfactory gyri; supplementary motor area; left superior temporal gyrus and temporal pole; bilateral caudate, putamen and pallidum. | -8 | 18 | -24 | 7.00 | 6.14 | 213520 |
|  | -4 | 44 | 20 | 6.91 | 6.08 |  |
|  | 40 | 22 | -8 | 6.89 | 6.06 |  |
| **Left middle** and **superior frontal** gyri and precentral | -46 | 20 | 40 | 6.12 | 5.51 | 1188 |
|  | -22 | 16 | 48 | 4.70 | 4.39 |  |
|  | -26 | 22 | 42 | 4.65 | 4.35 |  |
| **Bilateral precuneus**, middle and posterior **cingulate**. | -2 | -56 | 32 | 4.74 | 4.43 | 1199 |
|  | 6 | -54 | 32 | 4.58 | 4.29 |  |
|  | 0 | -34 | 32 | 4.40 | 4.14 |  |
| *bvFTD (tertile 3) vs HC* | | | | | | |
| **Bilateral anterior cingulate**, rectal gyrus, **medial frontal** gyrus, orbital gyri, olfactory gyrus. | -2 | 44 | -8 | 5.55 | 5.06 | 7632 |
|  | 6 | 46 | -4 | 5.38 | 4.93 |  |
|  | 10 | 42 | 22 | 5.28 | 4.85 |  |
| **Left** inferior frontal gyrus, **insula**, temporal pole. | -40 | 12 | -2 | 5.28 | 4.85 | 999 |
| ***ALZHEIMER’S DISEASE*** | | | | | | |
| *AD (tertile 1) vs HC* | | | | | | |
| **Bilateral precuneus**, **posterior** and middle **cingulate**, and cuneus. | 4 | -36 | 34 | 9.38 | Inf. | 5144 |
|  | 6 | -48 | 28 | 8.76 | 7.66 |  |
|  | -4 | -54 | 30 | 8.56 | 7.52 |  |
| **Left** middle and inferior temporal gyri, superior and **inferior parietal** lobules, supramarginal and **angular** gyri, precuneus, fusiform gyrus, superior and middle occipital gyri | -44 | -62 | 40 | 9.03 | 7.83 | 9951 |
|  | -52 | -66 | 30 | 8.93 | 7.77 |  |
|  | -34 | -52 | 40 | 8.29 | 7.33 |  |
| **Right** middle, superior, and **inferior temporal** gyri, angular, supramarginal gyri, superior and **inferior parietal** lobules. | 54 | -52 | 40 | 8.72 | 7.63 | 8819 |
|  | 64 | -30 | -20 | 8.46 | 7.45 |  |
|  | 46 | -66 | 44 | 8.11 | 7.20 |  |
| **Left middle** and superior **fronta**l gyri. | -42 | 10 | 40 | 6.08 | 5.65 | 2947 |
|  | -24 | 16 | 46 | 5.75 | 5.39 |  |
|  | -40 | 44 | 12 | 4.97 | 4.72 |  |
| **Right middle** and superior **frontal** gyri. | 40 | 24 | 46 | 5.31 | 5.01 | 2182 |
|  | 32 | 52 | 6 | 5.30 | 5.01 |  |
|  | 28 | 30 | 38 | 5.07 | 4.81 |  |
| *AD (tertile 2) vs HC* | | | | | | |
| **Left** inferior parietal lobule, **superior parietal lobule**, angular gyrus, **middle temporal** gyrus, and precuneus. | -32 | -72 | 44 | 5.67 | 5.31 | 1305 |
|  | -50 | -68 | 26 | 3.97 | 3.83 |  |
| **Right** angular gyrus, supramarginal gyrus, **precuneus**, superior and **inferior parietal lobule**, superior temporal gyrus, supramarginal gyrus, middle temporal gyrus. | 38 | -58 | 44 | 5.43 | 5.11 | 2272 |
|  | 50 | -54 | 40 | 5.39 | 5.08 |  |
|  | 38 | -74 | 42 | 5.10 | 4.84 |  |
| **Bilateral precuneus** and **posterior cingulate**. | -4 | -66 | 28 | 5.25 | 4.96 | 1545 |
|  | 6 | -56 | 30 | 4.61 | 4.41 |  |
|  | 0 | -32 | 40 | 4.44 | 4.25 |  |
| *AD (tertile 3) vs HC* | | | | | | |
| **Bilateral** precuneus, **posterior cingulate**, **superior** and **inferior parietal lobules**, angular gyri. | 10 | -66 | 56 | 5.02 | 4.76 | 6005 |
|  | 42 | -52 | 46 | 4.83 | 4.60 |  |
|  | -2 | -34 | 38 | 4.80 | 4.57 |  |
