# Supplementary material for: FDG-PET-based neural correlates of Addenbrooke’s cognitive examination III scores in Alzheimer’s disease and frontotemporal degeneration
Source: Front Psychol. 2023 Nov 16;14:1273608. doi: 10.3389/fpsyg.2023.1273608 (PMC10687370; doi:10.3389/fpsyg.2023.1273608)
Supplement: Supplementary file 6 [file Table_6.DOCX]

**~~Neural basis of the~~ FDG-PET-based neural correlates of Addenbrooke’s Cognitive Examination III scores in Alzheimer’s disease and frontotemporal degeneration**

María Nieves Cabrera-Martín (1) (*)

Pedro Nespral (1)

Maria Valles-Salgado (2)

Pablo Bascuñana (1)

Cristina Delgado-Alonso (2)

Alfonso Delgado-Álvarez (2)

Lucía Fernández-Romero (2)

Juan Ignacio López-Carbonero (2)

María Díez-Cirarda (2)

María José Gil-Moreno (2)

Jorge Matías-Guiu (2)

Jordi A Matias-Guiu (2)(*)

**Affiliations:**

- Department of Neurology. San Carlos Institute for Health Research (IdISSC), Universidad Complutense. Madrid, Spain.

- Department of Nuclear Medicine. San Carlos Institute for Health Research (IdISSC), Universidad Complutense. Madrid, Spain.

**Running title**: Neural basis of ACE-III

**(*) Corresponding Authors:**

Jordi A. Matias-Guiu. Department of Neurology. Hospital Clínico San Carlos. Prof. Martín Lagos St. 28040 Madrid (Spain). Phone number: +34913303511 +34676933312. E-mail: [jordimatiasguiu@hotmail.com](mailto:jordimatiasguiu@hotmail.com), [jordi.matias-guiu@salud.madrid.org](mailto:jordi.matias-guiu@salud.madrid.org)

María Nieves Cabrera-Martín. Department of Neurology. Hospital Clinico San Carlos. Prof Martin Lagos St. 28040 Madrid (Spain). Phone number: +34913303000. Madrid, Spain. E-mail: [mncabreram@hotmail.com](mailto:mncabreram@hotmail.com)

**ABSTRACT**

*Introduction*. The Addenbrooke’s Cognitive Examination III (ACE-III) is a brief test useful for neuropsychological assessment. Several studies have validated the test for the diagnosis of Alzheimer’s disease (AD) and frontotemporal dementia (FTD). In this study, we aimed to examine the metabolic correlates associated with the performance of ACE-III in AD and behavioral variant FTD.

*Methods*. We enrolled 300 participants in a cross-sectional study, including 180 patients with AD, 60 with behavioral FTD (bvFTD), and 60 controls. An ^18^F-Fluorodeoxyglucose positron emission tomography study was performed in all cases. Correlation between the ACE-III and its domains (attention, memory, fluency, language, and visuospatial) with the brain metabolism was estimated.

*Results*. The ACE-III showed distinct ~~different~~ neural correlates in bvFTD and AD, effectively capturing the most relevant regions involved in ~~with~~ these disorders. Neural correlates differed for each domain, especially in the case of bvFTD. Lower ACE-III scores ~~of the ACE-III~~ were associated with more advanced stages in both disorders. The ACE-III exhibited ~~showed~~ high discrimination between bvFTD vs HC, and between AD vs HC. Additionally, ~~and~~ it ~~also~~ was sensitive to detect hypometabolism in brain regions associated with bvFTD and AD.

*Conclusions*. Our study contributes to the knowledge of the brain regions associated with ACE-III, thereby facilitating its interpretation, and highlighting its suitability for ~~improving the interpretation of the test and suggesting its usefulness for~~ screening and monitoring. This study provides further ~~an additional~~ validation of ACE-III ~~the test~~ in the context of AD and FTD.

**Keywords:** Alzheimer’s disease; frontotemporal dementia; neuropsychological assessment; Addenbrooke’s Cognitive Examination; positron emission tomography.

**MAIN TEXT**

**Introduction**

The Addenbrooke’s Cognitive Examination III (ACE-III) is a screening cognitive test developed for diagnosing ~~the diagnosis of~~ cognitive disorders, ~~and, especially,~~ particularly ~~for~~ frontotemporal dementia (FTD) and Alzheimer’s disease (AD) (1). Initially, it ~~This~~ was ~~initially~~ validated as a screening tool for cognitive impairment in FTD and AD, but subsequently, it has shown ~~demonstrated a~~ high utility for the diagnosis of amnestic mild cognitive impairment, mild dementia of several types, early-onset dementia and dementia in the elderly (2-5). Furthermore, it has been validated in several languages and settings (6-7). ACE-III belongs to a new generation of brief cognitive tests designed to enhance the diagnostic capabilities of the ~~that aim to improve the diagnostic properties of~~ Mini-Mental State Examination (8). ~~However,~~ Importantly, ACE-III plays a dual function in cognitive examination: ~~on the one hand,~~ firstly, to screen for cognitive disorders; and, secondly, to obtain a cognitive profile (9). ~~The five cognitive domains assessed and the high correlation observed between these domains with standardized neuropsychological tests suggest that, beyond the screening, ACE-III could provide helpful information for the differential diagnosis between neurodegenerative diseases~~ The assessment covers five cognitive domains, and the strong correlations observed between these domains and standardized neuropsychological tests suggest that ACE-III could offer valuable information for differentiating between neurodegenerative diseases (5, 10-11).

The similarities and differences in the cognitive profile between bvFTD and AD is a matter of debate. While bvFTD is characterized by executive function impairment and relatively preserved episodic memory and visuospatial function (12), these distinctions are not absolute, as some patients with bvFTD may exhibit cognitive profiles similar to those seen in AD. It's worth noting that executive function is also affected in AD, and memory can be impaired in bvFTD. However, cognitive tests remain crucial for the diagnosis of these disorders across various levels of the healthcare system (13).

One of the main challenges in neuropsychological assessment is the interpretation of the findings. On one hand, as mentioned earlier, bvFTD and AD are associated with different cognitive profiles, but there is significant overlap (14). On the other hand, although each cognitive function is associated with more or less specific neural systems, multiple mechanisms and functional cognitive systems underlie the impairment of particular cognitive tasks. Current models of cognitive functions have revealed a more complex and distributed neural basis than previously assumed in traditional theories of localization of cognitive abilities. ~~there are multiple mechanisms and functional cognitive systems explaining the impairment of specific cognitive tasks and even current models of cognitive functions have shown more complex and distributed neural basis than previously expected in the traditional localization of cognitive abilities.~~ In this regard, the knowledge of the neural basis of cognitive tests has practical implications in clinical practice to improve the interpretation of findings and theoretical relevance for advancing our understanding ~~to advance in understanding~~ of brain-behavior relationships. However, relatively few works have explored ~~evaluated~~ this topic ~~for~~ in the context of brief cognitive tests (15). For the other brief tests, some studies have examined the capacity of MoCA to predict brain metabolism in patients with mild cognitive impairment due to AD and transient ischemic attack or lacunar stroke and hippocampal atrophy in patients with memory complaints (16,17). To the best of our knowledge, there are no previous studies analyzing ~~no previous studies have analyzed~~ the neural basis of the ACE-III. ~~so far.~~

In this study, we aimed to examine the neural correlates of ACE-III ~~in bvFTD and AD patients~~ in a large cohort of 300 participants with bvFTD and AD. This information may be valuable in interpreting the test and its cognitive domains. We also aimed to evaluate ~~examine~~ the diagnostic properties of ACE-III and determine ~~to define~~ the best cutoff points based on ~~considering~~ the clinical diagnosis and brain metabolism.

**Methods**

*Study design and population*

We enrolled 300 participants in a cross-sectional study, including 180 patients with AD, 60 with behavioral FTD, and 60 controls. Patients were diagnosed according to the current diagnostic criteria. Patients with bvFTD met the diagnostic criteria by Rascovsky et al., 2011 and had at least two years of follow-up confirming the diagnosis. Patients with AD were diagnosed according to the criteria by McKhann et al., 2011 and had neuroimaging and/or CSF confirmation (Aβ1-42, tau and phospho-tau) (12, 18). In cases of bvFTD, CSF biomarkers were used when considered clinically necessary. Cognitive assessment were carried out using a standardized protocol encompassing the following tests: digit span forward and backward; Corsi’s cubes forward and backward; Boston Naming Test; Trail Making Test parts A and B; Symbol Digit Modalities test; Free and Cued Selective Reminding Test; Rey-Osterrieth Complex Figure (copy and memory at 3 and 3 minutes); verbal fluency (semantic and letter); and Visual Object and Space Perception Battery (subtests object decision, progressive silhouettes, position discrimination, and number location). Additionally, global CDR for staging of AD patients and global CDR plus NACC FTLD rating for bvFTD were used (19,20).

Atypical variants of AD (consisting of 15 patients with posterior cortical atrophy and 104 with logogenic aphasia) and language variants of FTD (comprising 87 patients with non-fluent primary progressive aphasia and 43 with semantic aphasia) were excluded from this study. Only the ~~first~~ initial FDG-PET imaging of each patient was used ~~for this study~~. The recruitment took place at the Department of Neurology of the Hospital Clinico San Carlos (Madrid, Spain) between February 2015 and February 2021.

*Addenbrooke’s Cognitive Examination III*

All participants ~~were tested on the~~ underwent ACE-III testing, which includes ~~The ACE-III included~~ the following scores: total score (the sum of all items) and five domains: attention (scored between 0 and 18), memory (0-26), fluency (0-14), language (0-26), and visuospatial abilities (0-16) (1,4). The attention domain comprises the following tasks: time orientation (0-5), spatial orientation (0-5), repetition of 3 words (0-3), and serial subtractions (0-3). The memory domain ~~includes the~~ involves recalling of 3 ~~words~~ previously repeated words, ~~the~~ learning of a name and address of a person (0-7), ~~the~~ recalling ~~of~~ known historical and present facts (0-4), and ~~the~~ delayed recall~~ing~~ of the name and address (0-7). The fluency domain is assessed ~~with~~ through letter (words beginning with “p”) (0-7) and semantic (animals) verbal fluency (0-7). The language domain ~~comprises~~ encompasses the following tasks: understanding a set of physical commands (0-3); writing two complete sentences (0-2); repetition of complex words (0-2) and sentences (0-2); naming 12 drawings (0-12); semantic knowledge about the previous drawings (0-4); and reading of five stranger words to assess surface dyslexia (0-1). Finally, the visuospatial abilities domain ~~includes~~ comprises three visuoconstructive tasks (copying ~~of~~ two loops (0-1), a cube (0-2) and drawing a clock (0-5)), one visuospatial (counting a set of dots) (0-4), and one visuoperceptive task (recognizing four incomplete letters) (0-4).

The test was administered according to the guidelines and materials provided ~~given~~ for the Spanish-language version and can be accessed at ~~is available at~~ <https://frontierftd.org/> .

*Acquisition, preprocessing, and analysis of FDG-PET imaging*

PET-CT imaging was conducted using a Siemens Biograph True Point Platform equipped with a 6-slice detector. ~~Images were acquired in a PET-CT Siemens Biograph True Point Platform with a 6-slice detector.~~ Patients fasted for a minimum of ~~at least~~ 6 hours before receiving an average dose ~~the injection of a mean dose~~ of ^18^F-FDG of 185 MBq. A static PET image was acquired through a sinogram bed ~~after~~ 30 minutes after ~~of~~ administering the tracer, along with ~~and~~ the rest of the patient. CT parameters were set as follows: 130/40/1 (kVp/effective mAs/rotation time); slice thickness of 3 mm; reconstruction interval of 1.5 mm; and pitch of 0.75. Subsequently, iterative 3D image reconstruction was ~~then~~ performed using the True X method with two iterations and 21 subsets. The interval between cognitive assessment and FDG-PET was less than three months for all patients.

Statistical Parametric Mapping 12 (SPM12) (The Wellcome Trust Centre for Neuroimaging, Institute of Neurology, University College of London) was used for preprocessing and analysis of FDG-PET imaging (<https://www.fil.ion.ucl.ac.uk/spm/>). Images were first realigned and normalized to the Montreal Neurological Institute (MNI) and then smooth at 8 mm full width at half maximum. Global metabolism was introduced as a nuisance covariate.

Multiple regression analysis was used to study the positive correlation between ~~the~~ ACE-III scores and brain metabolism at a voxel level. Age, gender, and years of education were introduced as covariates. Furthermore, a two-sample T-test was used to define the regions impaired in each group against controls. These analyses were performed using SPM12, with a statistical threshold of FWE-corrected (cluster level) p-value <0.05 ~~was used as the statistical threshold~~. SPM maps are presented in neurological orientation, with the left hemisphere on the left-side, and the right hemisphere on the right). The MNI coordinate system was used for the localization of the regions in the standard space.

*Statistical analysis*

Statistical analysis was performed using the software IBM® SPSS Statistics 26.0. Descriptive results are shown as mean±standard deviation or frequency (percentage). Normality was assessed using the Kolmogorov-Smirnov test. To assess differences among the three groups (bvFTD, AD, HC), a Kruskall-Wallis test with posthoc Dunn analysis ~~procedure~~ was conducted. ~~to test for differences between the three groups (bvFTD, AD, HC).~~ A p-value <0.05 was considered statistically significant.

Receiver Operating Characteristic (ROC) curves were calculated to evaluate the discrimination between bvFTD vs HC, and AD vs HC using the ACE-III. ~~We also evaluated the discrimination between normal vs altered metabolism in the main regions associated with bvFTD and AD with the ACE-III.~~ Additionally, discrimination between normal and altered metabolism in the key regions associated with bvFTD and AD, as depicted in Supplementary Figure 1, was evaluated. An area under the curve (AUC) >0.7 was deemed acceptable. Youden’s index (YI) was ~~estimated to evaluate~~ calculated to determine the best cutoff points, and various diagnostic metrics, including sensitivity, specificity, positive and negative predictive values, positive and negative likelihood values, and Number Needed for Screening Utility (NNSU) were also computed (21).

**Results**

*ACE-III performance across groups*

Patients with bvFTD showed lower performance than controls in all ACE-III domains and in the total score ~~compared with controls~~. Similarly, patients with AD scored lower than controls in all the domains. Finally, patients with bvFTD scored lower than AD in fluency and language domain ~~compared with AD~~ (**Table 1** and **Supplementary Table 1**). The ~~area under the curve (AUC)~~ AUC for the discrimination between bvFTD and HC was 0.871 using the total score (p<0.001). The best cutoff was 81 (YI=0.683, Sensitivity 85%, Specificity 83.3%). The AUC for distinguishing AD and HC was 0.834 (p<0.001). In this case, the best cutoff was 85 (YI=0.528, Sensitivity 82.8%, Specificity 70%). All the metrics evaluating test accuracy are shown in **Table 2**. AUC and cutoff scores for each ACE-III domain are shown in **Supplementary Table 2.**

*Metabolic correlates of ACE-III in bvFTD*

The total score was positively correlated with the metabolism of the left superior, middle, and superior medial frontal gyrus.

The attention domain was correlated with the left superior and middle frontal gyri,

supplementary motor area, precentral gyrus and cingulate gyrus (anterior and mid parts).

The memory domain was associated with the metabolism of the left insula,

inferior, middle and superior frontal gyri, superior temporal gyrus, and anterior cingulate.

The fluency domain was correlated with two clusters, including the left frontal lobe (superior, middle, and inferior frontal gyri, precentral, middle and anterior cingulate) and extending to the left temporal (superior, middle and inferior temporal gyri), parietal (left inferior parietal lobule, angular and supramarginal gyri) and insula.

The language domain was not associated with any significant cluster at the prespecified threshold.

The visuospatial domain was correlated with the metabolism of the right superior and middle frontal gyri and the supplementary motor area (**Figure 1**).

Complete details about statistics are shown in **Supplementary Table 3**.

*Metabolic correlates of ACE-III in AD*

The total score was correlated with the metabolism of three large clusters involving the bilateral temporoparietal lobes and extending to some occipital regions.

The attention domain was correlated with bilateral superior, middle, and inferior temporal gyri, posterior cingulate, precuneus, inferior parietal lobule and bilateral angular and fusiform gyri.

The memory domain was correlated with bilateral superior, middle, and inferior temporal gyri, left parahippocampal gyrus and hippocampus, posterior and middle cingulate gyri, precuneus, and inferior parietal lobule.

The fluency domain was associated with a large cluster in the left hemisphere involving the superior, middle, and inferior temporal gyri, inferior and middle frontal gyri, angular and fusiform gyri, precuneus, anterior, superior and inferior parietal lobule, middle and posterior cingulate. It was also associated with a smaller cluster in the right temporal lobe.

The language domain was associated with the metabolism of the left hemisphere, especially with temporoparietal regions and left inferior and middle frontal gyri. It was also correlated with the right temporal lobe.

The visuospatial domain was correlated with bilateral temporal lobe, and left inferior parietal lobule, angular and supramarginal gyri, precuneus, posterior cingulate, lingual gyrus, and middle occipital gyrus (**Figure 2**).

Complete details about statistics are shown in **Supplementary Table 4**.

*bvFTD and AD metabolism and staging according to ACE-III*

Patients with bvFTD and AD were divided into three tertiles according to the ACE-III total score, and each group was compared with controls **(Figures 3 and 4**). Complete statistics are detailed in **Supplementary Table 5.**

*Estimation of cutoff scores based on FDG-PET imaging*

The AUC for the detection of hypometabolism in FTD-regions using the ACE-III (total score) was 0.805. The best cutoff was 80 (YI=0.567, sensitivity 78.95%, specificity 77.78%). The AUC for the detection of hypometabolism in AD-regions was 0.704. The best cutoff was 84 (YI=0.315, sensitivity 91.18%, specificity 40.29%). All the metrics evaluating test accuracy are shown in **Table 2** and **Supplementary Table 2** for domains scores..

**Discussion**

In this study, we aimed to evaluate the neural correlates of the ACE-III and its domains. We explored the relationship between each ACE-III score and resting-state brain metabolism ~~at rest~~ in two patient cohorts: ~~of patients with~~ those with bvFTD and AD. As expected, all ACE-III scores were significantly lower in these patient groups compared with HC, reflecting the global cognitive impairment that occurs as these neurodegenerative diseases progress. ~~that the process of neurodegeneration produces a global cognitive impairment as the disease advances.~~

In the comparison between AD and bvFTD, verbal fluency and language scores were lower in bvFTD, which is consistent with other studies (22). However, these findings are not replicated in all the FTD cohorts, due to the heterogeneity of these disorders, particularly FTD, and the time of assessment throughout the disease (1; 22-24). Verbal fluency in the ACE-III encompasses both semantic and letter fluency tasks, which draw on multiple cognitive domains, including memory and executive function. Therefore, it is impaired in both AD and bvFTD. However, individuals with more severe executive dysfunction may exhibit more pronounced deficits in both semantic and letter fluency, while patients with prominent episodic memory deficits generally impair only semantic fluency. In terms of the language domain, our study showed that it was more impaired in bvFTD than AD. The language domain involves naming abilities (12 out of 26 points), but also includes semantic tasks (5 points) or writing (2 points), among others. These tasks likely engage both language and executive networks, reflecting the heterogeneous yet significant language deficits previously described in other bvFTD cohorts (25-26). Despite these differences, it is important to recognize that there is a substantial overlap in scores between AD and bvFTD groups (as in other cognitive tests), and no single score alone is sensitive enough to reliably differentiate between these disorders. Additionally, bvFTD were more functionally impaired than AD patients.

Furthermore, our analysis revealed that the total ACE-III score was correlated with ~~several characteristic~~ brain regions typically impaired in the early stages of bvFTD and AD. ~~However,~~ Notably, there was a certain left hemisphere predominance ~~for the left hemisphere~~ in both cases. This may be attributed ~~is consistent~~ with the fact that the majority ~~of most of the items of the~~ ACE-III items involve ~~have a~~ verbal input or output. This would suggest that the test could be less sensitive to cases with predominant right hemisphere damage, which could be especially important in some variants of FTD in which asymmetry is more frequent than in AD. However, this left hemisphere bias could also be explained by the left hemisphere’s higher susceptibility to neurodegeneration in these disorders (27-31).

Interestingly, the ACE-III domains exhibited ~~showed~~ different neural correlates in bvFTD. Almost all the scores showed a left hemisphere predominance. Memory was associated with left frontal lobe function, and with a smaller cluster in the left temporal lobe. This confirms that frontal lobe dysfunction can produce memory impairment in bvFTD, supporting recent evidence that emphasizes episodic memory dysfunction in at least a subgroup of patients with bvFTD (32-33). Similarly, verbal fluency was more strongly associated with the left frontal lobe, extending to left temporal and parietal regions, according to previous literature (34). Interestingly, the visuospatial domain was correlated with the right superior and middle frontal gyri and supplementary motor area. This may be interpreted considering the role of prefrontal and premotor regions in visuospatial processing and the role of the right frontal cortex in planning of visuoconstructive tasks (35). The different neural correlates of the ACE-III domains could also be indirect evidence of the heterogeneity of this disorder, in which different cognitive profiles may be found.

We did not detect statistically significant associations with the language domain in the bvFTD. This could be explained because patients with language variants of FTD were specifically excluded. In this regard, the comparison between bvFTD and HC showed a relative sparing of brain regions more closely associated with language dysfunction in FTD (i.e. perisylvian regions and left anterior temporal lobe). ~~In addition,~~ Even after excluding patients with PPA, ~~patients~~ individuals with bvFTD show a ~~wide~~ range of mild language disorders, including semantic processing, comprehension skills, naming difficulties (36). This heterogeneity ~~could suggest the~~ may explain the lack of clear correlations with the ACE-III language domain.

Regarding AD, the different ACE-III domains were correlated with the main regions affected ~~impaired~~ in the ~~first~~ early stages of AD, comprising the bilateral temporoparietal lobes. These brain correlates ~~of the different~~ domains included encompassed some of the earliest regions involved in AD, such as the precuneus, posterior cingulate, and middle temporal gyrus. Interestingly, the attention domain was more closely associated related to with ~~the~~ parietal and temporal regions, rather than ~~but not~~ frontal regions. ~~On the one hand,~~ This alignment ~~this~~ is consistent with the inclusion of orientation and calculation tasks within ~~items in~~ this domain, as well as the known association between  ~~On the other hand, the association between~~ associative brain regions and attention and executive function in AD ~~is well-known~~ (37-38). In the visuospatial domain, the correlated regions also extended to the occipital lobe. ~~the case of the visuospatial domain, the regions correlated slightly extended also to the occipital lobe.~~

Overall, our findings support the notion of a distinct neural basis for ACE-III in bvFTD and AD. ~~these results support the differential neural basis of ACE-III between bvFTD and AD.~~ This highlights that specific cognitive scores within ACE-III may reflect dysfunction in various and heterogeneous brain regions. For instance, memory domain impairment may be associated with the left frontal lobe in cases with bvFTD or bilateral parieto-temporal lobe in AD. This should be considered when interpreting the results from each cognitive domain, in which the information of each domain should be put into the context of the other cognitive domains, behavior changes and functional status.

Another interesting result of our study is the demonstration that ACE-III ~~the test~~ is sensitive to changes in brain metabolism. This ~~confirms~~ suggests that ACE-III ~~may~~ can be used for monitoring and staging ~~these~~ patients during their follow-up. In this regard, upper tertiles of the test were associated with the earliest regions impaired in bvFTD and AD, ~~while~~ whereas lower tertiles were ~~are~~ linked to more advanced stages of each disorder. These findings are consistent with previous studies that have correlated ~~in which~~ ACE-III scores with ~~was related to~~ functional abilities (10), underscoring ACE-III as a reliable tool for patient follow-up and monitoring. ~~and suggest that ACE-III may be a reliable test for follow-up and monitoring.~~

In this study, we also proposed some ACE-III cutoffs based on brain metabolism. The determination of the best cutoffs for neuropsychological tests is a controversial issue. ~~There are several procedures to establish the best cutoffs for neuropsychological assessment.~~ The most used procedure is the estimation of ROC curves by comparing a diagnosis with a control group, and the calculation of a cutoff point according to the levels of sensitivity and specificity. However, fixed cutoffs may be less useful in some settings (e.g. lower levels of schooling) or disorders with a wide range of ages in the presentation. In this case, the collection of normative data may be a solution, and in the case of ACE-III it improved the diagnosis (39). However, in this case, the choice of the specific cutoff point (e.g. 1, 1.5 or 2 standard deviations below the norms) remains challenging. ~~is also difficult.~~ In this study, we examined the use of cutoff points based on biomarkers of brain function. In this regard, the good AUC values for detecting hypometabolism in bvFTD and AD regions support~~s~~ the utility of ACE-III, complementing ~~the~~ information from normative data and ~~previous~~ prior validation studies. It is worth noting that the AUC for detecting hypometabolism in the earliest regions impaired in AD was lower than in the regions impaired in bvFTD. Specifically, specificity and positive predictive values were limited when diagnosing AD versus HC. Utilizing more challenging and specific memory paradigms may offer improved diagnostic capacity in this context (40-41). ~~could be more useful in this regard (36-37).~~ In addition, patients with AD generally seek medical attention earlier than those with bvFTD (42), and in our cohort, patients with AD were diagnosed at earlier stages according to CDR. ~~tend to consult before patients with bvFTD (38).~~ Similarly, combining ACE-III with other tests examining cognitive functions early impaired in bvFTD (e.g. social cognition) could improve the diagnostic capacity (43-44). However, due to the difficulties in detecting early stages of AD and bvFTD, the AUC values based on brain metabolism are appropriate considering the time of administration of the test for a first assessment of patients. In our study, FDG-PET calculated cutoffs are ~~similar to~~ consistent with those estimated by comparing patients with ~~and~~ controls and are comparable ~~. In addition, these cutoffs are comparable~~ to those proposed by other ~~authors in several cohorts~~ researchers in various cohorts using clinical criteria (1, 23). This underscors the consistency of ACE-III in detecting AD and bvFTD and ~~confirms the consistency of the test to detect AD and bvFTD and~~ suggests a potential cross-cultural equivalence. Based on these findings, ACE-III should be regarded as a valuable screening tool, given its favorable sensitivity, but findings should be confirmed through additional cognitive tests or biomarkers due to its relatively low specificity, especially in the context of AD symptoms.

Several limitations of our study should be acknowledged. ~~Some limitations of our study should be taken into account.~~ Firstly, we ~~only based our study~~ focused our analysis on the total ACE-III score and domains scores. ~~of the ACE-III.~~ Exploring individual items might enhance the differentiation between disorders, as demonstrated in the case of primary progressive aphasia variants(44). ~~The analysis of the individual items could probably improve the differential diagnosis between disorders, as has been shown for primary progressive aphasia variants (41).~~ In addition, ~~the~~ qualitative assessment of ~~the~~ individual tasks could also provide valuable insights for diagnosis, as differences in error patterns between bvFTD and AD may exist (14). ~~additional and relevant information for diagnosis, because errors between bvFTD and AD may be different (14).~~ Secondly, due to the progressive nature ~~course~~ of neurodegenerative disorders and the involvement of several cognitive domains concurrently ~~at the same time, the isolation of~~ isolating cognitive functions for the analysis of their neural correlates is difficult. Due to the high correlation between the different domain scores, we could not control for the other scores to prevent the effect of collinearity. Thirdly, we have restricted our analysis to the most prototypical variants of AD (amnestic type) and FTD (behavioral variant). Future studies should aim to confirm these findings in the atypical variants of AD and FTD are necessary. Fourthly, pathological confirmation of the diagnosis was not available, and amyloid biomarkers were not available in all cases. However, the group comparison of FDG-PET imaging against controls confirmed the expected brain regions impaired in each disease.

In conclusion, our study contributes to the knowledge of the brain regions associated with ACE-III, improving the interpretation of the test and suggesting the usefulness of this test for screening and monitoring. These findings provide further evidence of the validity of ~~the~~ ACE-III for ~~the assessment~~ assessing ~~of~~ patients with bvFTD and AD in both clinical and research settings.

**References**

1-Hsieh S., Schubert S., Hoon C., Mioshi E., Hodges J.R. Validation of the Addenbrooke’s cognitive examination III in frontotemporal dementia and Alzheimer's disease. Dementia and Geriatric Cognitive Disorders 2013;36:242–250.

2-Elamin M, Holloway G, Bak TH, Pal S. The utility of the Addenbrooke’s Cognitive Examination version three in early-onset dementia. Dement Geriatr Cogn Disord 2016;41:9-15.

3-Jubb MT, Evans JJ. An investigation of the utility of the Addenbrooke’s Cognitive Examination III in the early detection of dementia in memory clinic patients aged over 75 years. Dement Geriatr Cogn Disord 2015;30:222-232.

4-Matías-Guiu JA, Fernández de Bobadilla R, Escudero G, Pérez-Pérez J, Cortés A, Morenas-Rodríguez E, Valles-Salgado M, Moreno-Ramos T, Kulisevsky J, Matías-Guiu J. Validation of the Spanish version of Addenbrooke’s Cognitive Examination III for diagnosing dementia. Neurología 2015;30:545–551.

5-Matías-Guiu JA, Cortés-Martínez A, Valles-Salgado M, Rognoni T, Fernández-Matarrubia M, Moreno-Ramos T, Matías-Guiu J. Addenbrooke’s Cognitive Examination III: diagnostic utility for mild cognitive impairment and dementia and correlation with standardized neuropsychological tests. Int Psychogeriatrics 2017;29:105-113.

6-Charerboon T, Jaisin K, Lerthattasilp T. The Thai version of the Addenbrooke’s Cognitive Examination III. Psychiatry Investig 2016;13:571-573.

7-Wang BR, Ou Z, Gu XH, Wei CS, Xu J, Shi JQ. Validation of the Chinese version of Addenbrooke’s Cognitive Examination III for diagnosing dementia. Int J Geriatr Psychiatry 2017. Doi:10.1002/gps.4680.

8-Matias-Guiu JA, Valles-Salgado M, Rognoni T, Hamre-Gil F, Moreno-Ramos T, Matias-Guiu J. Comparative diagnostic accuracy of the ACE-III, MIS, MMSE, MoCA, and RUDAS for screening of Alzheimer’s disease. Dement Geriatr Cogn Disord 2017;43:237-246.

9-Matías-Guiu JA, Fernández-Bobadilla R, Cortés-Martínez A. Addenbrooke’s Cognitive Examination III: a neuropsychological test useful to screen and obtain a cognitive profile. Neurologia 2016. Doi:10.1016/j.nrl.2016.06.014.

10-So M, Foxe D, Kumfor F, Murray C, Hsieh S, Savage G, Ahmed RM, Burrell JR, Hodges JR, Irish M, Piguet O. Addenbrooke’s Cognitive Examination III: psychometric characteristics and relations to functional ability in dementia. J Int Neuropsychol Soc 2018;24:854-863.

11-Zarrella GV, Kay CD, Gettens K, Sherman JC, Colvin MK. Addenbrooke’s Cognitive Examination-Third Edition predicts neuropsychological test performance. J Neuropsychiatry Clin Neurosci 2023;35:178-183.

12-Rascovsky K, Hodges JR, Knopman D, Kramer JH, Neuhaus J, van Swieten JC, Seelaar H, Dopper EG, Onyike CU, Hillis AE, Josephs KA, Boeve BF, Kertesz A, Seeley WW, Rankin KP, Johnson JK, Gorno-Tempini ML, Rosen H, Prioleau-Latham CE, Lee A, Kipps CM, Lillo P, Piguet O, Rohrer JD, Rossor MN, Warren JD, Fox NC, Galasko D, Salmon DP, Black SE, Mesulam M, Weintraub S, Dickerson BC, Diehl-Schmid J, Pasquier F, Deramecourt V, Lebert F, Pijnenburg Y, Chow TW, Manes F, Grafman J, Cappa SF, Freedman M, Grossman M, Miller BL. Sensitivity of revised diagnostic criteria for the behavioural variant of frontotemporal dementia. Brain 2011; 134: 2456-2477.

13-Cruz de Souza L, Hosogi ML, Machado TH, Carthery-Goulart MT, Yassuda MS, Smid J, Barbosa BJAP, Schilling LP, Balthazar MLF, Frota NAF, Vale FAC, Caramelli P, Bertolucci PHF, Chaves MLF, Brucki SMD, Nitrini R, Bahia S, Takada LT. Diagnosis of frontotemporal dementia: recommendations of the Scientific Department of Cognitive Neurology and Aging of the Brazilian Academy of Neurology. Dement Neuropsychol 2022;16:40-52.

14-Musa G, Slachevsky A, Muñoz-Neira C, Mendez-Orelana C, Villagra R, González-Billault C, Ibáñez A, Hornberger M, Lillo P. Alzheimer’s disease or frontotemporal dementia? Review of key points toward an accurate clinical and neuropsychological diagnosis. J Alzheimers Dis 2020;73:833-848.

15-Paul R, Lane EM, Tate DF, Heaps J, Romo DM, Akbudak E, Niehoff J, Conturo TE. Neuroimaging signatures and cognitive correlates of the Montreal Cognitive Assessment screen in a nonclinical elderly sample. Arch Clin Neuropsychol 2011;26:454-460.

16-Zukotynski K, Gaudet V, Kuo PH, Adamo S, Goubran M, Scott CJM, Bocti C, Borrie M, Chertkow H, Frayne R, Hsiung R, Laforce R Jr, Noseworthy MD, Prato FS, Sahlas DJ, Smith EE, Sossi V, Thiel A, Soucy JP, Tardif JC, Black SE. The use of random forests to identify brain regions on amyloid and FDG PET associated with MoCA score. Clin Nucl Med 2020;45:427-433.

17- Ritter A, Hawley N, Banks SJ, Miller JB. The association between Montreal Cognitive Assessment Memory Scores and hippocampal volume in a neurodegenerative disease sample. J Alzheimers Dis 2017;58:695-699.

18-McKhann GM, Knopman DS, Chertkow H, et al. The diagnosis of dementia due to Alzheimer's disease: recommendations from the National Institute on Aging‐Alzheimer's Association workgroups on diagnostic guidelines for Alzheimer's disease. Alzheimer's & dementia 2011;7: 263-269.

19-Morris JC. The Clinical Dementia Rating (CDR): current version and scoring rules. Neurology 1993;43:2412-2414.

20-Miyagawa T, Brushaber D, Syrjanen J, Kremers W, Fields J, Forsberg LK, Heuer HW, Knopman D, Kornak J, Boxer A, Rosen HJ, Boeve BF, Appleby B, Bordelon Y, Bove J, Brannelly P, Caso C, Coppola G, Dever R, Dheel C, Dickerson B, Dickinson S, Dominguez S, Domoto-Reilly K, Faber K, Ferrell J, Fishman A, Fong J, Foroud T, Gavrilova R, Gearhart D, Ghanzanfari B, Ghoshal N, Goldman JS, Graff-Radford J, Graff-Radford N, Grant I, Grossman M, Haley D, Hsiung R, Huey E, Irwin D, Jones D, Jones L, Kantarci K, Karydas A, Kaufer D, Kerwin D, Kraft R, Kramer J, Kukull W, Litvan I, Lucente D, Lungu C, Mackenzie I, Maldonado M, Manoochehri M, McGinnis S, McKinley E, Mendez MF, Miller B, Multani N, Onyike C, Padmanabhan J, Pantelyat A, Pearlman R, Petrucelli L, Potter M, Rademakers R, Ramos EM, Rankin K, Rascovsky K, Roberson ED, Rogalski E, Sengdy P, Shaw L, Tartaglia MC, Tatton N, Taylor J, Toga A, Trojanowski JQ, Wang P, Weintraub S, Wong B, Wzsolek Z. Utility of the global CDR® plus NACC FTLD rating and development of scoring rules: data from the ARTFL/LEFFTDS Consortium. Alzheimers Dement 2020;16:106-117.

21-Larner AJ. New unitary metrics for dementia test accuracy studies. Prog Neurol Psychiatry 2019; 23:21-25.

22-Siri S, Benaglio I, Frigerio A, Binetti G, Cappa SF. A brief neuropsychological assessment for the differential diagnosis between frontotemporal dementia and Alzheimer’s disease. Eur J Neurol 2001;8:125-132.

23-Reul S, Lohmann H, Wiendl H, Duning T, Johnen A. Can cognitive assessment really discriminate early stages of Alzheimer’s and behavioural variant frontotemporal dementia at initial clinical presentation? Alzheimers Res Ther 2017;9:61.

24-Bruno D, Slachevsky A, Fiorentino N, Rueda DS, Bruno G, Tagle AR, Olavarria L, Flores P, Lillo P, Roca M, Torralva T. Argentinian/Chilean validation of the Spanish language version of Addenbrooke’s Cognitive Examination III for diagnosing dementia. Neurologia 2020;35:82-88.

25-Geraudie A, Battista P, Garcia AM, Allen IE, Miller ZA, Gorno-Tempini ML, Montembeault M. Speech and language impairments in behavioral variant frontotemporal dementia: a systematic review. Neurosci Biobehav Rev 2021;131:1076-1095.

26-Hardy CJD, Buckley AH, Downey LE, Lehmann M, Zimmerer V, Varley R, Crutch SJ, Rohrer JD, Warrington EK, Warren JD. The language profile of behavioral variant frontotemporal dementia. J Alzheimers Dis 2016;50:359-371.

27-Donix M, Burggren AC, Scharf M, Marschner K, Suthana NA, Siddarth P, Krupa AK, Jones M, Martin-Harris L, Ercoli LM, Miller KJ, Werner A, von Kummer R, Sauer C, Small GW, Holthoff VA, Bookheimer SY. APOE associated hemispheric asymmetry of entorhinal cortical thickness in aging and Alzheimer’s disease. Psychiatry Res 2013; 214:212-220.

28-Shi F, Liu B, Zhou Y, Yu C, Jiang T. Hippocampal volumen and asymmetry in mild cognitive impairment and Alzheimer’s disease: meta-analyses of MRI studies. Hippocampus 2009;19:1055-1064.

29-Thompson PM, Hayashi KM, de Zubicaray G, Janke AL, Rose SE, Semple J, Herman D, Hong MS, Dittmer SS, Doddrell DM, Toga AW. Dynamics of gray matter loss in Alzheimer’s disease. J Neurosci 2003;23:994-1005.

30-Whitwell JL, Xu J, Mandrekar J, Boeve BF, Knopman DS, Parisi JE, Senjem ML, Dickson DW, Petersen RC, Rademakers R, Jack CR Jr, Josephs KA. Frontal asymmetry in behavioral variant frontotemporal dementia: clinicoimaging and pathologenetic correlates. Neurobiol Aging 2013;32:636-639.

31-Rohrer JD. Structural brain imaging in frontotemporal dementia. Biochim Biophys Acta 2012;1822:325-332.

32-Fernandez-Matarrubia M, Matias-Guiu JA, Cabrera-Martin MN, Moreno-Ramos T, Valles-Salgado M, Carreras JL, Matias-Guiu J. Episodic memory dysfunction in behavioral variant frontotemporal dementia: a clinical and FDG-PET study. J Alzheimers Dis 2017;57:1251-1264.

33-Poos JM, Jiskoot LC, Papma JM, van Swieten JC, van den Berg E. Meta-analytic review of memory impairment in behavioral variant frontotemporal dementia. J Int Neuropsychol Soc 2018;24:593-605.

34-Delgado-Álvarez A, Cabrera-Martin MN, Pytel V, Delgado-Alonso C, Matias-Guiu J, Matias-Guiu JA. Design and verbal fluency in Alzheimer’s disease and Frontotemporal dementia: clinical and metabolic correlates. J Int Neuropsychol Soc 2022;28:947-962.

35-Delgado-Álvarez A, Cabrera-Martin MN, Valles-Salgado M, Delgado-Alonso C, Gil MJ, Díez-Cirarda M, Matias-Guiu J, Matias-Guiu JA. Neural basis of visuospatial tests in behavioral variant frontotemporal dementia. Front Aging Neurosci 2022;14:963751.

36-Geraudie A, Diax Rivera M, Montembeault M, Garcia AM. Language in behavioral variant frontotemporal dementia: another stone to be turned in Latin America. Front Neurol 2021;12:702770.

37-Habeck C, Risacher S, Lee GJ, Glymour MM, Mormino E, Mukherjee S, Kim S, Nho K, DeCarli C, Saykin AJ, Crane PK; Alzheimer’s Disease Neuroimaging Initiative. Relationship between baseline brain metabolism measured using [18F]FDG PET and memory and executive function in prodromal and early Alzheimer’s disease. Brain Imaging Behav 2012;6:568-583.

38- Matías-Guiu JA, Cabrera-Martín MN, Valles-Salgado M, Pérez-Pérez A, Rognoni T, Moreno-Ramos T, Carreras JL, Matías-Guiu J. Neural basis of cognitive assessment in Alzheimer disease, amnestic mild cognitive impairment, and subjective memory complaints. Am J Geriatr Psychiatry 2017. Doi: 10.1016/j.agp.2017.02.002.

39-Matías-Guiu J.A., Fernández-Bobadilla R., Fernández-Oliveira A., Valles-Salgado M., Rognoni T., Cortés-Martínez A., Moreno-Ramos T, Kulisevsky J, Matías-Guiu J. Normative data for the Spanish version of the Addenbrooke’s Cognitive Examination III. Dementia and Geriatric Cognitive Disorders 2016;41:243-250.

40-Curiel-Cid RE, Matias-Guiu JA, Loewenstein DA. A review of novel Cognitive Challenge Tests for the assessment of preclinical Alzheimer’s disease. Neuropsychology 2022. Doi:10.1037/neu0000883.

41-Valles-Salgado M, Cabrera-Martin MN, Curiel-Cid RE, Delgado-Alvarez A, Delgado-Alonso C, Gil-Moreno MJ, Matias-Guiu J, Loewenstein DA, Matias-Guiu JA. Neuropsychological, metabolic, and connectivity underpinnings of semantic interference deficits using the LASSI-L. J Alzheimers Dis 2022;90:823-840.

42-Ellajosyula R, Narayanan J, Hegde S, Kamath V, Murgod U, Easwaran V, Seetharam R, Srinivasan M, Watson P. Delay in the diagnosis of dementia in urban India: role of dementia subtype and age at onset. Int J Geriatr Psychiatry 2022. Doi:10.1002/gps.5843.

43-Dodich A, Cerami C, Cappa SF, Marcone A, Golzi V, Zamboni M, Giusti MC, Iannaccone S. Combined socio-behavioral evaluation improves the differential diagnosis between the behavioral variant of frontotemporal dementia and Alzheimer’s disease: in search of neuropsychological biomarkers. J Alzheimers Dis 2018; 61:761-772.

44-García-Gutiérrez F, Delgado-Álvarez A, Delgado-Alonso C, Díaz-Álvarez J, Pytel V, Valles-Salgado M, Gil MJ, Hernández-Lorenzo L, Matias-Guiu J, Ayala JL, Matias-Guiu JA. Diagnosis of Alzheimer’s disease and behavioural variant frontotemporal dementia with machine learning-aided neuropsychological assessment using feature engineering and genetic algorithms. Int J Geriatr Psychiatry 2021;11:37.

45-Foxe D, Hu A, Cheung SC, Ahmed RM, Cordato NJ, Devenney E, Hwang YT, Halliday GM, Mueler N, Leyton CE, Hodges JR, Burrell JR, Irish M, Piguet O. Utility of the Addenbrooke’s Cognitive Examination III online calculator to differentiate the primary progressive aphasia variants. Brain Commun 2022;4:fcac161.

**Conflict of interest statement:**

The authors declare that they have no disclosures to report.

# Author Contributions

MNCM: Conceptualization, Visualization, Data curation, Formal analysis, Investigation, Methodology, Funding acquisition, Writing – original draft, Writing – review & editing.

PN: Data curation, Investigation, Writing – review & editing.

MVS: Data curation; Investigation, Writing – review & editing.

PB: Investigation, Writing – review & editing.

CDA: Data curation; Investigation, Writing – review & editing.

ADA: Data curation; Investigation, Writing – review & editing.

LFR: Data curation; Investigation, Writing – review & editing.

JILP: Data curation; Investigation, Writing – review & editing.

MDC: Data curation; Investigation, Writing – review & editing.

MJG: Investigation, Writing – review & editing.

JMG: Conceptualization, Visualization, Funding acquisition, Investigation, Supervision, Writing –review & editing.

JAMG: Conceptualization, Visualization, Data curation, Formal analysis, Funding acquisition, Investigation, Methodology, Supervision, Writing – original draft, Writing – review & editing.

**Funding:**

JAMG is supported by Instituto de Salud Carlos III through the project

INT20/00079 (co-funded by European Regional Development Fund “A way to

make Europe”). MVS is supported by Instituto de Salud Carlos III through a

predoctoral contract PFIS (FI20/000145) (co-funded by European Regional

Development Fund “A way to make Europe”). MDC is funded by a Sara

Borrell postdoctoral fellowship from the Instituto de Salud Carlos III (CD22/00043)

(co-funded by European Regional Development Fund “A way to make Europe”). PB is

funded by a Miguel Servet postdoctoral position from the Instituto de Salud

Carlos III (CP21/00020) (co-funded by European Regional Development Fund “A way to make Europe”)

| **Table 1**. Main demographic characteristics and ACE-III performance across groups. | | | | | |
| --- | --- | --- | --- | --- | --- |
|  | | bvFTD  (n=60) | AD  (n=180) | HC  (n=60) | H ~~F~~/X^2^ (p-value) |
| Age | | 71.13±7.97 | 72.84±6.33 | 71.03±5.59 | 6.23 (0.044) |
| Sex (women) | | 24 (40.0%) | 97 (53.9%) | 36 (60%) | 5.24 (0.073) |
| Years of education | | 10.12±4.49 | 10.11±5.00 | 11.40±4.14 | 3.88 (0.143) |
| CDR global^*^ | 0.5 | 17 (28.3%) | 101 (56.1%) | - | 21.36 (<0.001) |
|  | 1 | 28 (46.7%) | 64 (35.6%) |  |  |
|  | 2 | 13 (21.7%) | 15 (53.6%) |  |  |
|  | 3 | 2 (3.3%) | 0 (0%) |  |  |
| ACE-III (total score)^a,b^ | | 64.80±18.01 | 69.52±16.97 | 87.13±8.22 | 70.62 (<0.001) |
| ACE-III (attention)^a,b^ | | 13.88±3.67 | 13.96±3.39 | 16.83±1.17 | 45.02 (<0.001) |
| ACE-III (memory)^a,b^ | | 13.80±5.88 | 13.68±5.67 | 20.57±4.23 | 61.17 (<0.001) |
| ACE-III (fluency)^a,b,c^ | | 5.75±3.78 | 8.23±3.55 | 11.08±1.89 | 61.32 (<0.001) |
| ACE-III (language)^a,b,c^ | | 18.88±4.95 | 21.04±4.69 | 23.90±2.66 | 40.89 (<0.001) |
| ACE-III (visuospatial)^a,b^ | | 12.08±3.04 | 12.49±2.99 | 14.70±1.49 | 35.82 (<0.001) |
| ~~ANOVA with Tukey~~ Kruskall-Wallis with post-hoc analysis (adjusted by Bonferroni) showed statistically significant differences after Bonferroni correction between bvFTD vs HC (a), AD vs HC (b), and bvFTD vs AD (c).  ^*^CDR global for AD and global CDR plus NACC FTLD for bvFTD. | | | | | |

| **Table 2**. Metrics for test accuracy using ACE-III (total score) | | | | |
| --- | --- | --- | --- | --- |
|  | Diagnosis | | Hypometabolism in FDG-PET | |
|  | AD vs HC | bvFTD vs HC | AD-regions | bvFTD-regions |
| AUC | 0.834 | 0.871 | 0.704 | 0.805 |
| Best cutoff | 85 | 81 | 84 | 80 |
| Sensitivity | 82.8% | 85.0% | 91.1% | 78.9% |
| Specificity | 70% | 83.3% | 40.2% | 77.7% |
| PPV | 89.2% | 83.6% | 20.1% | 76.2% |
| NPV | 57.53% | 84.7% | 96.5% | 80.3% |
| LR+ | 2.76 | 5.08 | 1.53 | 3.53 |
| LR- | 0.24 | 0.18 | 0.22 | 0.27 |
| NNSU | 0.87 | 0.70 | 1.75 | 0.81 |
| AUC: area under the curve; PPV: positive predictive value; NPV: negative predictive value; LR+: positive likelihood ration; LR-: negative likelihood ratio; NNSU: Number Needed for Screening Utility. | | | | |

**FIGURE LEGEND**

-**Figure 1.** Voxel-based brain mapping analysis showing the correlation between ACE-III scores and brain metabolism in bvFTD (FWE-corrected p-value <0.05). ACE-III total score is represented in *red,* ACE-III attention in *blue*, ACE-III fluency in *violet*, ACE-III language in *yellow*, ACE-III memory in *green*, and ACE-III visuospatial in *orange*.

**-Figure 2.** Voxel-based brain mapping analysis showing the correlation between ACE-III scores and brain metabolism in AD (FWE-corrected p-value <0.05). ACE-III total score is represented in *red,* ACE-III attention in *blue*, ACE-III fluency in *violet*, ACE-III language in *yellow*, ACE-III memory in *green*, and ACE-III visuospatial in *orange*.

**-Figure 3**. Voxel-based brain mapping analysis showing the regions with a lower brain metabolism in each of the tertile groups of bvFTD against HC (FWE-corrected p-value <0.05). ACE-III scores of each group are shown.

**-Figure 4.** Voxel-based brain mapping analysis showing the regions with a lower brain metabolism in each of the tertile groups of AD against HC (FWE-corrected p-value <0.05). ACE-III scores of each group are shown.

-**Supplementary Figure 1**. Regions included in the definition of hypometabolism in AD (*red*) and bvFTD (*blue*).
