# Supplementary figures and images for: FDG-PET-based neural correlates of Addenbrooke’s cognitive examination III scores in Alzheimer’s disease and frontotemporal degeneration
Source: Front Psychol. 2023 Nov 16;14:1273608. doi: 10.3389/fpsyg.2023.1273608 (PMC10687370; doi:10.3389/fpsyg.2023.1273608)

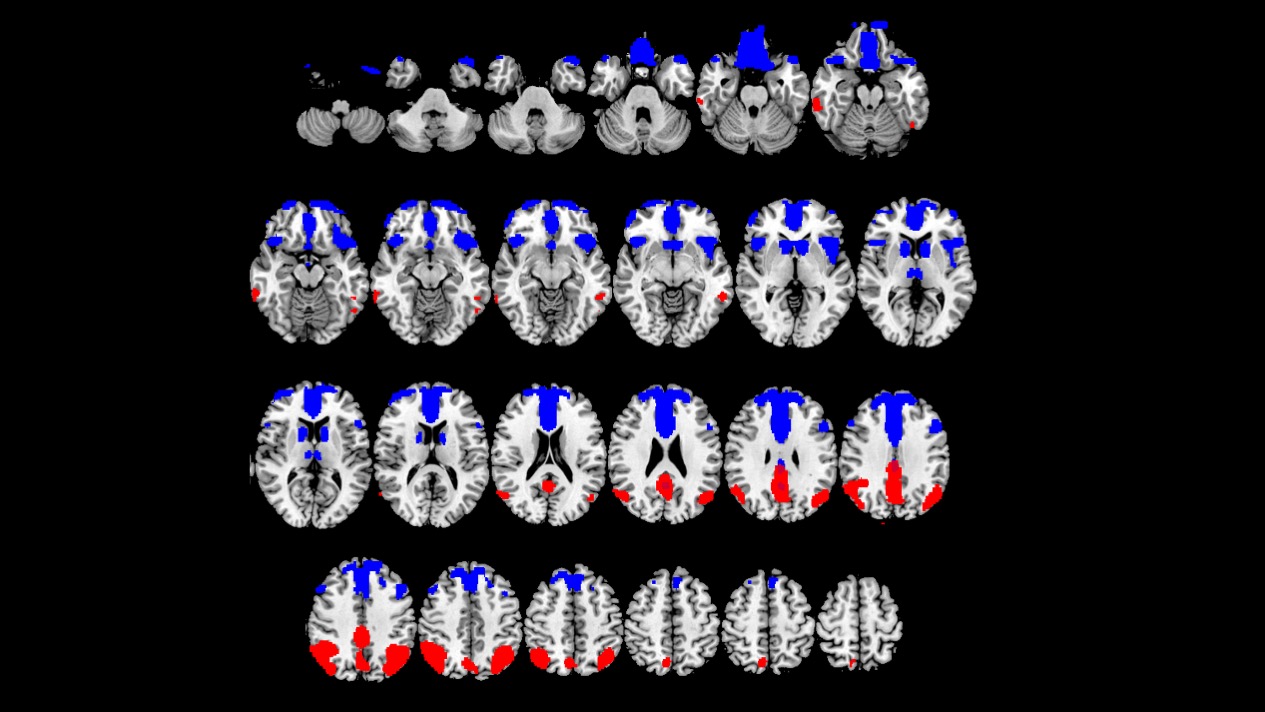

Supplement: Supplementary file 7 [file Image_1.JPEG]
